# In Silico Characterization and Determination of Gene Expression Levels Under Saline Stress Conditions in the Zinc Finger Family of the C1-2i Subclass in *Chenopodium quinoa* Willd

**DOI:** 10.3390/ijms26062570

**Published:** 2025-03-13

**Authors:** Andrea Alvarez-Vasquez, Luz Lima-Huanca, Roxana Bardales-Álvarez, Maria Valderrama-Valencia, Sandro Condori-Pacsi

**Affiliations:** Escuela de Biología, Facultad de Ciencias Biológicas, Universidad Nacional de San Agustín de Arequipa, Arequipa 04001, Peru; llima@unsa.edu.pe (L.L.-H.); rbardales@unsa.edu.pe (R.B.-Á.); mvalderramav@unsa.edu.pe (M.V.-V.); scondorip@unsa.edu.pe (S.C.-P.)

**Keywords:** *Chenopodium quinoa*, ZAT genes, transcription factors, C2H2 zinc fingers, salt stress, gene expression, transcriptional regulation, stress tolerance

## Abstract

Quinoa (*Chenopodium quinoa*) is recognized for its tolerance to abiotic stress, including salinity, and its recent genome sequencing has facilitated the study of the mechanisms underlying this adaptation. This study focused on characterizing the ZAT genes of the C2H2 subfamily in quinoa, evaluating their expression under saline stress. Eight ZAT genes were identified and analyzed in silico using genomic databases and bioinformatics tools, assessing their conserved domains, cis-regulatory motifs, and physicochemical characteristics. Additionally, germination assays, hydroponic cultivation, and gene expression analyses via qPCR were performed on halotolerant (UNSA_VP033) and halosensitive (UNSA_VP021) accessions exposed to different NaCl concentrations. The genes *CqZAT4* and *CqZAT6* showed high expression in the halotolerant accession under saline stress, correlating with increased dry matter, root length, and water retention. In contrast, the halosensitive accession exhibited lower tolerance, with significant reductions in these metrics. Promoter analysis revealed cis-elements associated with hormonal and stress responses. ZAT genes play a key role in quinoa’s response to saline stress, with *CqZAT4* and *CqZAT6* standing out in the halotolerant accession. These findings could drive the development of more resilient varieties, contributing to agricultural sustainability in saline soils.

## 1. Introduction

Quinoa (*Chenopodium quinoa* Willd.) is a facultative halophytic allotetraploid plant (2n = 4x = 36) belonging to the Amaranthaceae family. It was domesticated approximately 7000 years ago in the highlands of the central Andes in South America [1,2]. Considered a staple food by Andean civilizations due to its adaptability to extreme conditions, quinoa has recently resurged globally, standing out for its exceptional nutritional profile, rich in high-quality proteins, essential amino acids, fiber, vitamins, and minerals, in addition to being gluten-free, positioning it as a “superfood” [1]. Its production, led by countries like Peru and Bolivia, has expanded globally, becoming a key economic resource for agricultural communities and a dietary alternative in the face of current nutritional challenges [3].

Agronomically, quinoa is notable for its adaptability to adverse conditions such as arid climates, poor soils, and high salinity, characteristics that make it relevant in sustainable agriculture [4,5]. These qualities, along with the recent sequencing of its genome, have allowed the identification of genetic mechanisms responsible for these adaptations, facilitating the development of more resistant and productive varieties in stressful environments [2]. As a strategic crop in the context of climate change, quinoa not only offers a viable solution for marginal agricultural regions but also contributes to global food security and environmental conservation [3].

Soil salinity is one of the environmental problems affecting millions of crops, especially in arid and semi-arid zones, and threatens to further reduce agricultural land. This phenomenon, exacerbated by intensive agricultural practices and inadequate irrigation use, generates the accumulation of mineral salts in the soils, significantly reducing their fertility and productivity [4,6]. Climate change intensifies this problem by worsening moisture loss and increasing the concentration of salts in the soil. In fact, it is estimated that almost 10.7% of the world’s land surface is already affected by salinity, endangering global food security and further threatening the availability of arable land [4,6,7]. With projections indicating that half of cultivable land could be at risk by 2050, it is crucial to develop sustainable strategies, such as the genetic improvement of crops tolerant to saline stress, to face this crisis and guarantee agricultural and food sustainability [6,7,8]. Understanding the mechanisms of quinoa adaptation will enhance its cultivation potential, contributing to global food security.

Transcription factors (TFs) regulate gene expression by modifying transcription levels according to endogenous and exogenous stimuli [7]. The zinc finger protein (ZFP) gene family is crucial in plants, participating in processes such as: (1) plant development, (2) transcriptional regulation, and (3) apoptosis. C2H2-type ZFP proteins are common in eukaryotes and fulfill essential functions by specifically binding to DNA [9,10].

The ZAT proteins of the ZFP-C2H2 family, C1-2i subclass, are significant in the response to salt stress. Species such as *Gossypium hirsutum* [11,12], *Fragaria × ananassa* [13], *Nelumbo nucifera* [14], *Malus domestica* [15], *Arabidopsis thaliana* [16], *Populus trichocarpa* [17], *Ipomoea batatas* [18], and *Oryza sativa* [19], have been identified where these genes are involved in salt tolerance (NaCl). Overexpression studies of these genes in *Arabidopsis thaliana* enabled the reduction of reactive oxygen species [12,15], malondialdehyde content [15], increased antioxidant enzyme activity [20], abscisic acid (ABA), and gibberellin (GA8) content [20], promoting salt stress tolerance. Additionally, these genes are regulated by protein kinases [15] and ABA-dependent or independent pathways [14,21], which activate or inhibit a set of genes that help mediate the salt stress response. However, these genes are not only involved in salt stress response but also in responses to drought, cold, and osmotic stress [22].

The ZAT proteins of the ZFP-C2H2 family, subclass C1-2i, are characterized by two zinc finger domains containing invariant QALGGH motifs [23]. Their N-terminal region includes a B-box motif, functioning as a nuclear localization signal, followed by an L-box motif involved in protein-protein interactions. The C-terminal region contains a short hydrophobic DLN-box with an EAR motif [24].

In quinoa, various ZAT genes have been identified; however, their functionality remains unstudied. The objective of this work is to characterize in silico the ZAT proteins of the ZFP-C2H2 family, subclass C1-2i, and to determine their gene expression levels under different salt concentrations in *Chenopodium quinoa* Willd. This study can significantly contribute to improving quinoa cultivation under salinity stress conditions, enhancing its resilience and yield in such environments. In the long term, the results could facilitate the development of better-adapted quinoa varieties, supporting agricultural sustainability and global food security by enabling more efficient cultivation in saline soils.

## 2. Results

### 2.1. Identification of CqZAT Genes and Analysis of Conserved Domains, Motifs, and Gene Structure

Sequence similarity searches in *C. quinoa* were conducted using the C1-2i C2H2 members from *A. thaliana* on the Phytozome and NCBI databases. A total of 50 protein sequences from *C. quinoa* were retrieved. Among these candidates, only 8 proteins contained the two conserved domains characteristic of C2H2-type ZFPs, a defining feature of the C1-2i subclass. The identified proteins were assigned the prefix “Cq” (for *Chenopodium quinoa*), followed by the group name (ZAT) and an Arabic numeral corresponding to their registered number. These proteins were further characterized using data from the CDD-NCBI database, enabling precise identification of the number and position of domains in each CqZAT protein.

**Figure 1:**
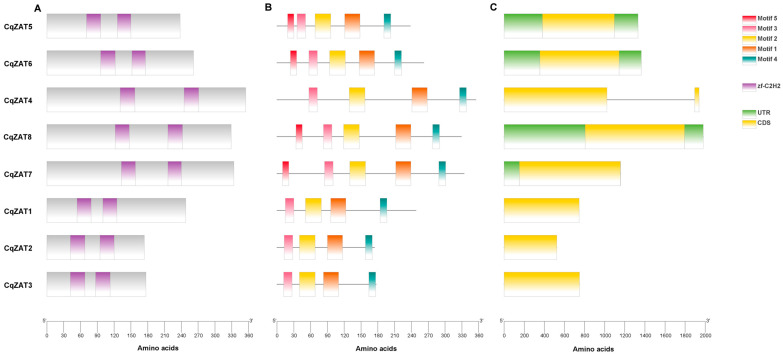
Conserved Domains, Motifs, and Gene Structure of CqZATs. (**A**) Conserved Domains of CqZAT Proteins. All proteins were analyzed using CDD-NCBI to identify conserved domains. The analysis revealed that all proteins contain zf-C2H2 domains (purple rectangles), corresponding to the C2H2 zinc finger domains. (**B**) Conserved Motif Structure of CqZAT Genes: Using MEME, five conserved motifs were identified. Colored boxes represent conserved motifs, while gray lines indicate non-conserved sequences. (**C**) Gene Structure of CqZATs: Yellow rectangles represent coding regions (CDS), green rectangles correspond to untranslated regions (UTRs), and black bars indicate introns.

Figure 1A illustrates the domain analysis, showing that all CqZAT proteins contain two zf-C2H2 domains, confirming their membership in the zinc finger protein family. The zf-C2H2 domains span 24 to 26 amino acids, indicating high conservation across all proteins.

Figure 1B presents the conserved motif analysis of CqZAT proteins, performed using the online MEME motif discovery tool. Five motifs were identified: motifs 1 and 2 correspond to zinc fingers, motif 3 corresponds to the L-box, motif 4 to the DLN-box, and motif 5 to the B-box. Among the analyzed proteins, CqZAT5, CqZAT6, CqZAT7, and CqZAT8 contained all five motifs, while CqZAT1, CqZAT2, CqZAT3, and CqZAT4 contained the first four motifs. Additionally, serine (S) was identified as the most frequent amino acid in these sequences (Table A1).

Figure 1C shows the structural analysis of ZAT genes, revealing that most have a single exon, with the exception of CqZAT4, which has a structure comprising two exons and one intron.

### 2.2. Physicochemical Characterization of CqZAT Genes

The physicochemical characterization (Table 1), conducted using the ExPASy web server, revealed that CqZAT proteins range in length from 174 to 355 amino acids. Their molecular weights vary from 19.38 to 38.12 kDa, and their isoelectric points (pI) range between 5.84 and 9.48. To assess structural stability, the aliphatic index of the sequences was determined, yielding values between 58.13 and 82.15.

Regarding cellular localization, predictions suggest that these proteins are primarily localized in the nucleus, but may also be present in other cellular compartments, such as the cytoplasm, chloroplasts, and periplasm. This indicates diverse and specialized functions within the cell.

### 2.3. Sequence Alignment and Phylogenetic Analysis

**Figure 2:**
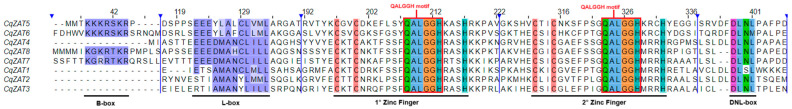
Multiple Sequence Alignment of CqZAT Amino Acid Sequences. Conserved amino acid residues across all sequences are highlighted in various colors. The QALGGH motif is marked with red boxes. The B-box (nuclear localization signal, NLS), DLN-box, L-box, and the two zinc finger domains are indicated with solid black bars. Solid blue bars denote abbreviated regions of amino acids within the sequences.

In Figure 2, the multiple sequence alignment analysis in Clustal Omega shows that all protein sequences in *C. quinoa* contain two conserved C2H2-type zinc finger (ZF) domains (CX2-4CX3FX5LX2HX3-5H). Each ZF domain carries the QALGGH motif, which is plant-specific. They also exhibit an EAR transcriptional repression motif, featuring the DLNL sequence (DLN-box) at the C-terminal end. Among the eight *C. quinoa* protein sequences, all harbor four domains: the L-box, two zinc finger domains, and the DLN-box. However, the B-box domain is present only in four sequences: CqZAT5, CqZAT6, CqZAT7, and CqZAT8.

**Figure 3:**
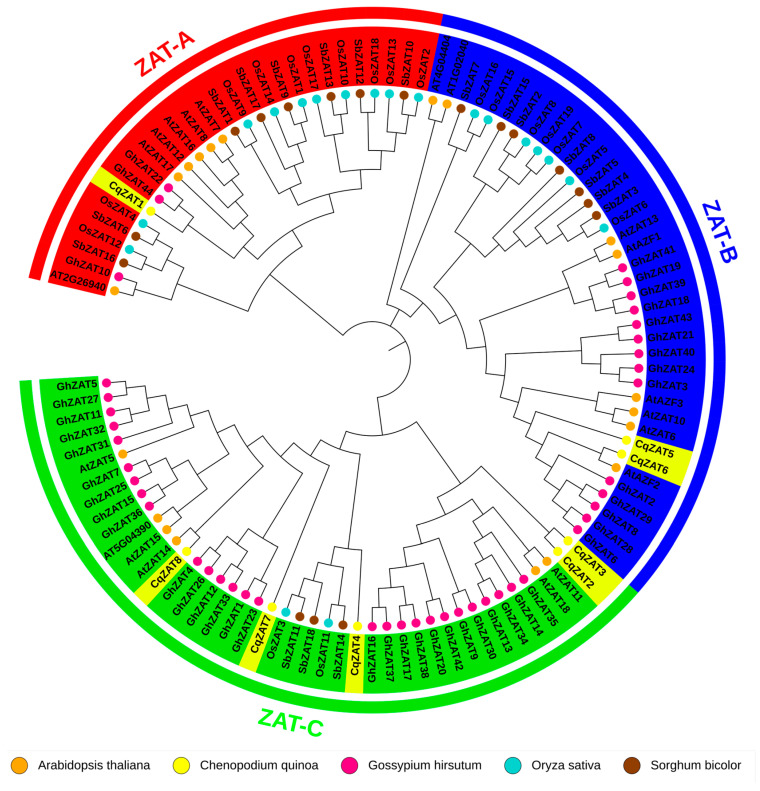
Phylogenetic Tree of ZAT Genes in *Chenopodium quinoa*, *Arabidopsis thaliana*, *Gossypium hirsutum*, *Oryza sativa*, and *Sorghum bicolor*. The phylogenetic tree was constructed in MEGA X using the Neighbor-Joining method, the Jones-Taylor-Thornton (JTT) substitution model, and a gamma (G) distribution. A bootstrap value of 1000 was applied. The genes are grouped into three subfamilies: ZAT-A (red), ZAT-B (blue), and ZAT-C (green). Colored dots represent different species: orange (*Arabidopsis thaliana*), yellow (*Chenopodium quinoa*), pink (*Gossypium hirsutum*), light blue (*Oryza sativa*), and brown (*Sorghum bicolor*).

All ZAT proteins from quinoa (*C. quinoa*), Arabidopsis (*A. thaliana*), cotton (*G. hirsutum*), rice (*Oryza sativa*), and sorghum (*Sorghum bicolor*) were aligned to generate a phylogenetic tree using the Neighbor-Joining method (Jones-Taylor-Thornton [JTT] model and gamma [G] distribution) in MEGA X. Figure 3 shows the resulting phylogenetic tree, which is divided into three main evolutionary groups or subfamilies: ZAT-A, ZAT-B, and ZAT-C. Within each group, the ZAT genes are more closely related to one another, suggesting that they may share similar or complementary functions in different species. The *C. quinoa* ZAT genes (CqZAT1 to CqZAT8) are distributed among these three subfamilies. ZAT-A includes CqZAT1, ZAT-B includes CqZAT5 and CqZAT6, and ZAT-C comprises CqZAT2, CqZAT3, CqZAT4, CqZAT7, and CqZAT8. Notably, CqZAT5 and CqZAT6 cluster together with *A. thaliana* (AtAZF2, AtZAT6, AtZAT10, and AtAZF3) and *G. hirsutum* (GhZAT6) genes that have been reported to confer salt stress tolerance.

### 2.4. Cis-Regulatory Element Analysis of CqZAT Promoter Regions

**Figure 4:**
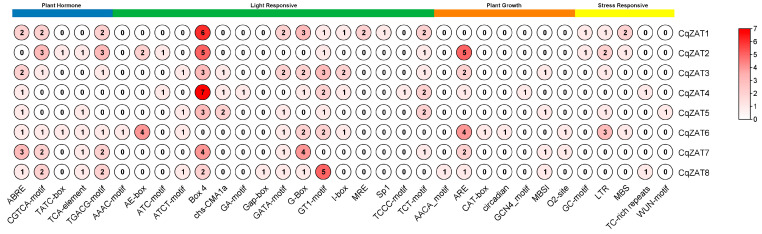
Cis-Element Analysis of CqZAT Promoter Regions. The colored bars at the top indicate the groups into which the cis-elements were categorized. The frequency of each cis-element is displayed as a number within the circle. The color intensity of the circles is proportional to the score, with the corresponding relationship between numbers and colors depicted using a color scale in the right panel.

To gain deeper insights into the potential functions of CqZAT genes, a 2000 bp upstream promoter region was analyzed for each CqZAT gene using the PlantCARE online server. As shown in Figure 4, a total of 33 cis-elements (Table A2) were identified and classified into four groups: hormone response, light response, plant growth, and stress response. The hormone response group contains five elements associated with abscisic acid response (ABRE), methyl jasmonate (MeJA) response (CGTCA-motif and TGACG-motif), gibberellin response (TATC-box), and salicylic acid response (TCA-element). The light response group has 16 elements, making it the largest group. The plant growth group contains seven elements involved in negative endosperm-specific expression (AACA_motif), anaerobic induction (ARE), meristem expression (CAT-box), circadian control (circadian), endosperm expression (GCN4_motif), flavonoid biosynthesis regulation (MBSI), and zein metabolism regulation (O2-site). Finally, the stress response group includes five elements involved in low oxygen availability (GC-motif), low temperature (LTR), drought (MBS), defense and stress response (TC-rich repeats), and wounding (WUN-motif). These findings demonstrate that CqZAT genes participate broadly in multiple physiological and biochemical plant activities and are critical for responding to diverse environmental cues and stress conditions.

### 2.5. Germination Percentage and Identification of Halotolerant and Halosensitive Accessions

**Figure 5:**
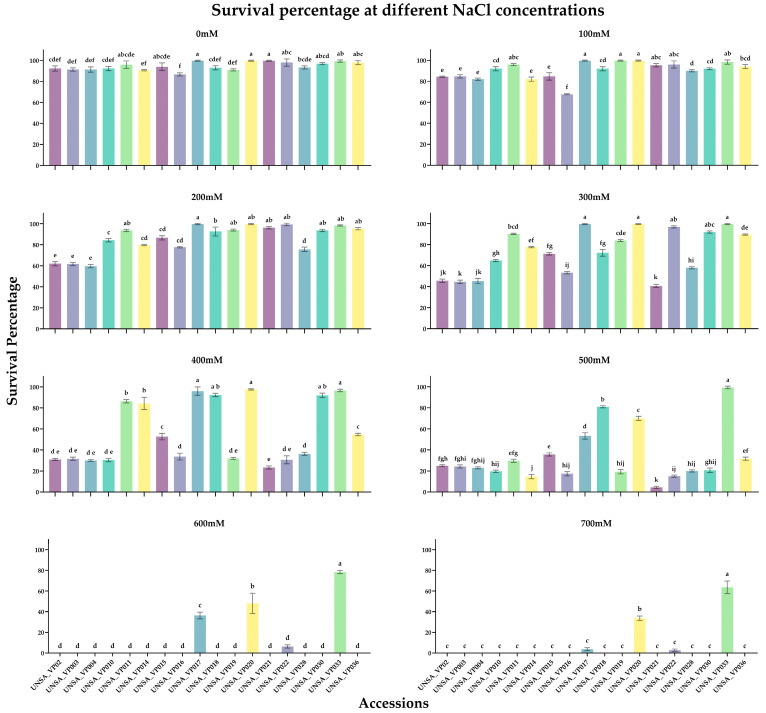
Survival Percentage of Different Accessions Under Increasing NaCl Concentrations (0 mM, 100 mM, 200 mM, 300 mM, 400 mM, 500 mM, 600 mM, and 700 mM). The data were analyzed using a one-way ANOVA, followed by Tukey’s post hoc test. Different letters indicate statistically significant differences between groups (*p* < 0.05).

Figure 5 displays the 18 accessions analyzed to determine their survival percentage under varying salt concentrations.

At NaCl concentrations of 0 mM, 100 mM, and 200 mM, all accessions exhibited high tolerance percentages, exceeding 85%, 65%, and 60%, respectively. At 300 mM, the accession UNSA_VP021 showed the lowest survival percentage (41%), followed by UNSA_VP003 and UNSA_VP004 (45% each), and UNSA_VP002 (46%). The remaining accessions showed survival rates above 50%, with UNSA_VP017, UNSA_VP020, and UNSA_VP033 achieving 100%.

At 400 mM, nine accessions maintained a survival percentage above 50%, with UNSA_VP017, UNSA_VP018, UNSA_VP020, UNSA_VP030, and UNSA_VP033 exceeding 90%. UNSA_VP021 recorded the lowest survival percentage (23%).

At 500 mM, 14 accessions had survival percentages below 37%, with UNSA_VP021 showing the lowest survival rate (4%). Meanwhile, UNSA_VP017, UNSA_VP018, UNSA_VP020, and UNSA_VP033 surpassed 58%, with UNSA_VP033 reaching a remarkable survival rate of 99%.

At 600 mM and 700 mM, only three accessions survived (UNSA_VP017, UNSA_VP020, and UNSA_VP033). UNSA_VP033 displayed survival percentages of 78% and 64% at these concentrations, respectively, while the other accessions showed no survival.

### 2.6. Determination of Halotolerant and Halosensitive Accessions in a Hydroponic System

**Figure A1:**
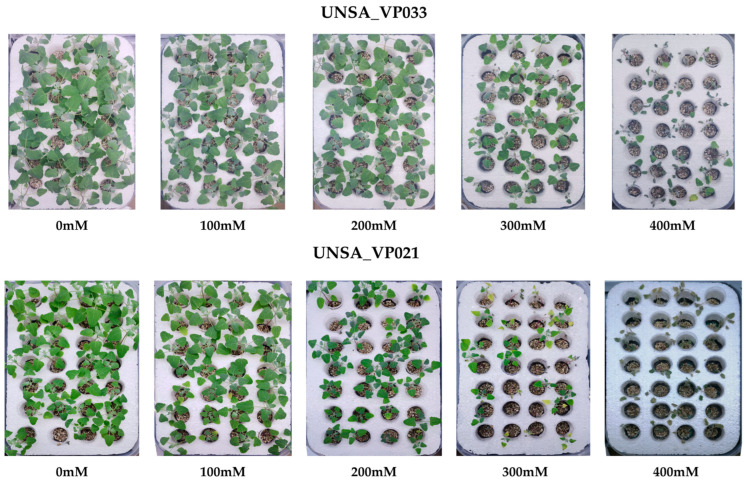
Comparison of Growth in *Chenopodium quinoa* Accessions UNSA_VP033 and UNSA_VP021 Under Different NaCl Concentrations (0 mM, 100 mM, 200 mM, 300 mM, and 400 mM).

Accessions UNSA_VP033 and UNSA_VP021 were transferred to a hydroponic system and exposed to different NaCl concentrations (0 mM, 100 mM, 200 mM, 300 mM, and 400 mM) to evaluate their tolerance capacity and median lethal dose. Plants without chlorosis were counted to determine survival. The results indicate that the median lethal dose for UNSA_VP033 was 300 mM, while for UNSA_VP021 it was 200 mM (Figure A1).

### 2.7. Determination of Dry Matter Percentage

**Figure 6:**
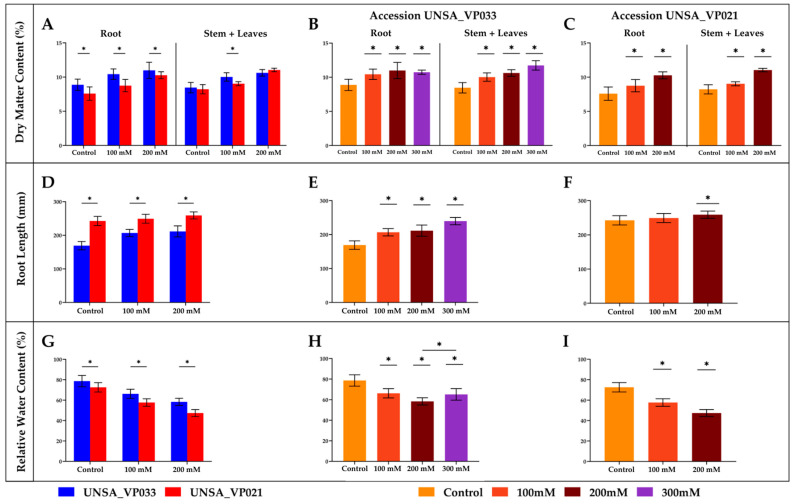
Effect of Saline Stress on *Chenopodium quinoa* Accessions UNSA_VP033 and UNSA_VP021 Under Different NaCl Concentrations (0 mM, 100 mM, 200 mM, and 300 mM). (**A**) Percentage of dry matter (%DM) in root and aerial parts of UNSA_VP033 and UNSA_VP021 accessions at NaCl concentrations of 0 mM, 100 mM, and 200 mM. (**B**) Percentage of dry matter (%DM) in root and aerial parts of UNSA_VP033 at NaCl concentrations of 0 mM, 100 mM, 200 mM, and 300 mM. (**C**) Percentage of dry matter (%DM) in root and aerial parts of UNSA_VP021 at NaCl concentrations of 0 mM, 100 mM, and 200 mM. (**D**) Root length (in mm) of UNSA_VP033 and UNSA_VP021 accessions at NaCl concentrations of 0 mM, 100 mM, and 200 mM. (**E**) Root length (in mm) of UNSA_VP033 at NaCl concentrations of 0 mM, 100 mM, 200 mM, and 300 mM. (**F**) Root length (in mm) of UNSA_VP021 at NaCl concentrations of 0 mM, 100 mM, and 200 mM. (**G**) Relative water content (RWC) of UNSA_VP033 and UNSA_VP021 accessions at NaCl concentrations of 0 mM, 100 mM, and 200 mM. (**H**) Relative water content (RWC) of UNSA_VP033 at NaCl concentrations of 0 mM, 100 mM, 200 mM, and 300 mM. (**I**) Relative water content (RWC) of UNSA_VP021 at NaCl concentrations of 0 mM, 100 mM, and 200 mM. Images (**A**,**D**,**G**) were evaluated, while the remaining images were analyzed through ANOVA followed by Dunnett’s post-hoc test. Asterisks (*) indicate significant differences between groups at a significance level of *p* < 0.05. The data were analyzed using Student’s *t*-test (Images **A**,**D**,**G**) and a two-way ANOVA, followed by Dunnet’s post-hoc test (Images **B**,**C**,**E**,**F**,**H**,**I**). Asterisks (*) indicate significant differences between groups at a significance level of *p* < 0.05.

In Figure 6A–C, a significant increase (*p* < 0.05) in dry matter percentage was observed in both root and aerial parts of accessions UNSA_VP033 and UNSA_VP021 under all treatments compared to the control. UNSA_VP033 exhibited a higher dry matter percentage under saline stress conditions.

In Figure 6D–F, root length is shown for both accessions. Root length significantly increased (*p* < 0.05) in UNSA_VP033 under all treatments compared to the control. For UNSA_VP021, a significant increase (*p* < 0.05) was observed at 200 mM compared to the control.

### 2.8. Determination of Relative Water Content (RWC)

Figure 6G–I display the relative water content in accessions UNSA_VP033 and UNSA_VP021. In both accessions, relative water content significantly decreased (*p* < 0.05) as salt concentration increased. UNSA_VP033 maintained higher water levels compared to UNSA_VP021.

### 2.9. Chlorophyll and Carotenoid Content

**Figure 7:**
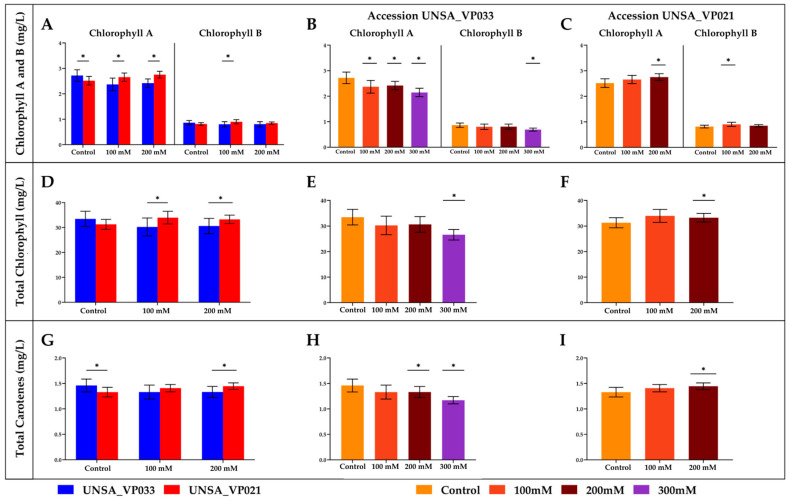
Effect of Saline Stress on *Chenopodium quinoa* Accessions UNSA_VP033 and UNSA_VP021 Under Different NaCl Concentrations (0 mM, 100 mM, 200 mM, and 300 mM). (**A**) Chlorophyll A, chlorophyll B, total chlorophyll, and total carotenoid content in UNSA_VP033 and UNSA_VP021 accessions under NaCl concentrations of 0 mM, 100 mM, and 200 mM. (**B**) Chlorophyll A and chlorophyll B content in UNSA_VP033 under NaCl concentrations of 0 mM, 100 mM, 200 mM, and 300 mM. (**C**) Chlorophyll A and chlorophyll B content in UNSA_VP021 under NaCl concentrations of 0 mM, 100 mM, and 200 mM. (**D**) Total chlorophyll content in UNSA_VP033 and UNSA_VP021 under NaCl concentrations of 0 mM, 100 mM, and 200 mM. (**E**) Total chlorophyll content in UNSA_VP033 under NaCl concentrations of 0 mM, 100 mM, 200 mM, and 300 mM. (**F**) Total chlorophyll content in UNSA_VP021 under NaCl concentrations of 0 mM, 100 mM, and 200 mM. (**G**) Total carotenoid content in UNSA_VP033 and UNSA_VP021 under NaCl concentrations of 0 mM, 100 mM, and 200 mM. (**H**) Total carotenoid content in UNSA_VP033 under NaCl concentrations of 0 mM, 100 mM, 200 mM, and 300 mM. (**I**) Total carotenoid content in UNSA_VP021 under NaCl concentrations of 0 mM, 100 mM, and 200 Mm. The data were analyzed using Student’s *t*-test (Images **A**,**D**,**G**) and a two-way ANOVA, followed by Dunnet’s post-hoc test (Images **B**,**C**,**E**,**F**,**H**,**I**). Asterisks (*) indicate significant differences between groups at a significance level of *p* < 0.05.

In Figure 7A–C, significant changes (*p* < 0.05) in chlorophyll A and B levels were observed in UNSA_VP033 and UNSA_VP021 accessions under different saline treatments (100 mM, 200 mM, and 300 mM) compared to the control. In UNSA_VP033, chlorophyll A content significantly decreased (*p* < 0.05) as salt concentration increased, while chlorophyll B showed a significant decrease (*p* < 0.05) only at 300 mM compared to the control. In UNSA_VP021, the chlorophyll A content significantly increased (*p* < 0.05) at 200 mM compared to the control, while chlorophyll B content showed a significant increase (*p* < 0.05) at 100 mM.

Figure 7D–F display total chlorophyll content. In UNSA_VP033, the total chlorophyll content significantly decreased (*p* < 0.05) at 300 mM compared to the control. UNSA_VP021 exhibited a significant increase (*p* < 0.05) in total chlorophyll content at 200 mM compared to the control.

Figure 7G–I show total carotenoid content. In UNSA_VP033, the carotenoid levels significantly decreased (*p* < 0.05) at 100 mM and 200 mM compared to the control. In UNSA_VP021, a significant increase (*p* < 0.05) in carotenoid content was observed at 200 mM compared to the control.

### 2.10. Relative Expression of ZAT Genes in C. quinoa

**Figure 8:**
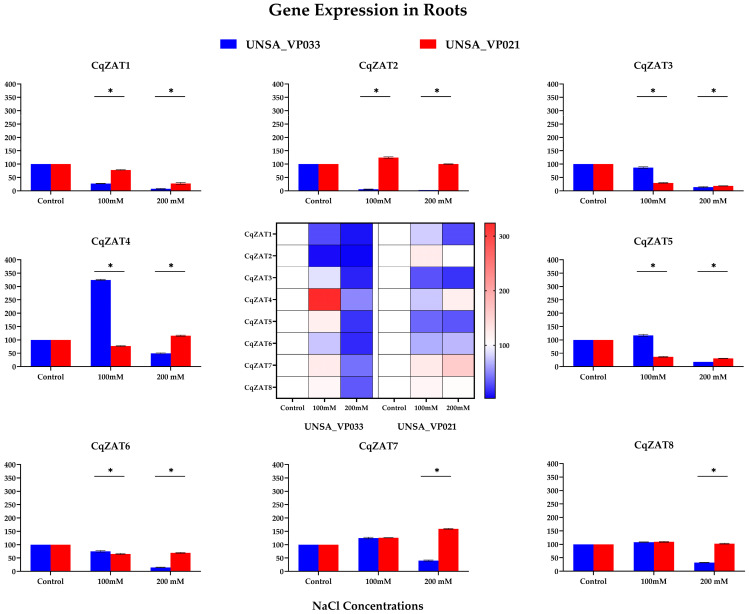
Relative Gene Expression of the CqZAT Family in Roots of *Chenopodium quinoa* Accessions UNSA_VP033 and UNSA_VP021 Under Saline Stress Conditions. The data were analyzed using Student’s *t*-test. Asterisks (*) indicate significant differences between groups at a significance level of *p* < 0.05.

Figure 8 presents a heatmap illustrating gene expression levels for all genes in both accessions. In UNSA_VP033, gene CqZAT4 shows a strong red intensity at 100 mM NaCl, indicating high expression compared to other genes.

Genes CqZAT1, CqZAT2, CqZAT3, CqZAT5, and CqZAT6 exhibited a significant decrease (*p* < 0.05) in expression under saline stress in both accessions. Conversely, CqZAT4 displayed significantly higher expression (*p* < 0.05) in UNSA_VP033 at 100 mM NaCl compared to the control and other treatments. For CqZAT7 and CqZAT8, significant differences (*p* < 0.05) in expression were observed at 200 mM compared to the control (Figures S1–S7).

**Figure 9:**
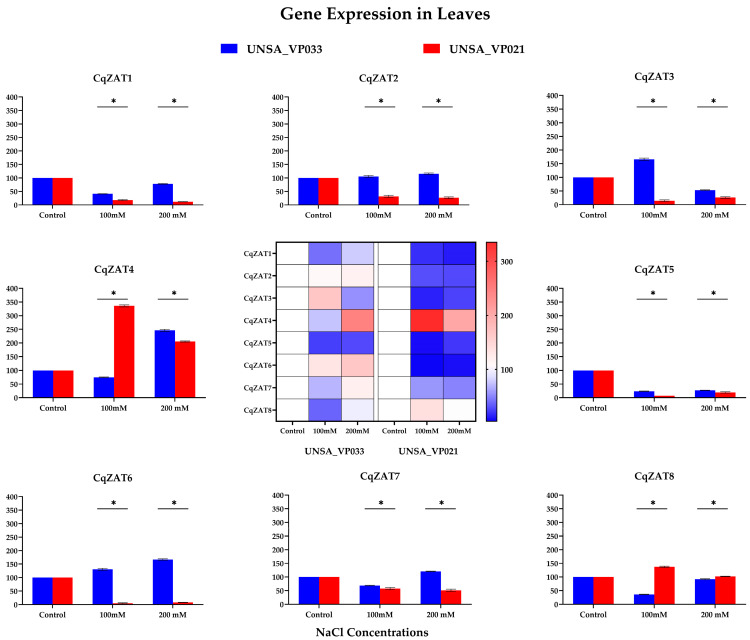
Relative Gene Expression of the CqZAT Family in Leaves of *Chenopodium quinoa* Accessions UNSA_VP033 and UNSA_VP021 Under Saline Stress Conditions. The data were analyzed using Student’s *t*-test. Asterisks (*) indicate significant differences between groups at a significance level of *p* < 0.05.

Figure 9 displays the heatmap in the center, illustrating the expression levels of all genes in both accessions. In UNSA_VP021, gene CqZAT4 shows a strong red intensity at 100 mM NaCl, indicating high gene expression compared to other genes.

Genes CqZAT1, CqZAT2, CqZAT5, and CqZAT7 exhibited a significant decrease (*p* < 0.05) in gene expression under saline stress conditions in both accessions. Gene CqZAT3 displayed a significant increase (*p* < 0.05) in UNSA_VP033 at 100 mM NaCl compared to the control, but showed a significant decrease (*p* < 0.05) at 200 mM. Gene CqZAT4 showed a significantly higher expression (*p* < 0.05) in UNSA_VP021 at 100 mM NaCl compared to the control and other treatments.

Gene CqZAT6 exhibited a significant difference (*p* < 0.05) in expression between treatments in accession UNSA_VP033 compared to UNSA_VP021, where CqZAT6 showed a greater decrease (*p* < 0.05) under treatment conditions relative to the control. Finally, gene CqZAT8 showed a significant difference (*p* < 0.05) in expression at 100 mM NaCl in UNSA_VP021 compared to the control (Figures S8–S14).

## 3. Discussion

### 3.1. In Silico Characterization of ZAT Proteins from the C2H2 Zinc Finger Subclass C1-2i in Chenopodium quinoa

The characterization of ZAT genes in *Chenopodium quinoa* identified eight proteins with C2H2 domains, characteristic of zinc finger transcription factors. These domains, with lengths ranging from 24 to 26 amino acids, are highly conserved among CqZAT proteins (Figure 1A), reflecting strong evolutionary conservation to maintain structure and function. Similar conservation of C2H2 domains has been reported in species such as *A. thaliana* [16] and *I. batatas* [18], where C2H2-ZFPs regulate genes associated with abiotic stress responses. Conservation in other species, including *Populus* [17] and *Gossypium* [11,12], reinforces the essential and conserved role of these proteins in environmental stress responses.

Conserved motif analysis using MEME (Figure 1B) and sequence alignment (Figure 2) revealed five primary motifs in CqZAT proteins: C2H2-type zinc finger domains, L-box, DLN-box (also known as EAR or DLNL motif), and B-box. The zinc fingers, including the plant-specific QALGGH motif, are essential for DNA binding and transcriptional regulation, which are critical for stress responses and development [9,10,24,25]. The L-box facilitates protein-protein interactions, enabling ZAT proteins to form functional complexes with other regulatory proteins. The B-box serves as a nuclear localization signal, and the DLN-box indicates that ZAT genes may act as transcriptional repressors in response to environmental conditions [16,26,27]. Among the identified proteins, CqZAT5, CqZAT6, CqZAT7, and CqZAT8 contain all five motifs, while CqZAT1, CqZAT2, CqZAT3, and CqZAT4 lack the B-box. This motif conservation is also observed in species like *Populus* [17] and *N. nucifera* [14], where it is associated with specific subgroups of the C2H2 family, highlighting their adaptive role in abiotic stress.

The predominance of serine (S) residues in the sequences (Table A1) suggests potential regulation via phosphorylation, a key process in stress signaling. This aligns with studies by Liu et al. [28], demonstrating that phosphorylation of AtZAT6 by MPK6 enhances germination under stress conditions. This evidence supports the hypothesis that serine phosphorylation may activate signaling cascades during adverse conditions, enhancing plant adaptability.

Structurally, most CqZAT genes have a single exon (Figure 1C), except for CqZAT4, which has two exons. This structure facilitates rapid transcription during stress, consistent with patterns observed in *I. batatas* [18], *Solanum lycopersicum* [29], *Populus* [30], and *Gossypium* [12]. The presence of an intron in CqZAT4 suggests functional diversification in response to environmental pressures [31].

Physicochemical analysis (Table 1) revealed protein sizes of 174–355 amino acids and molecular weights of 19.38–38.12 kDa, aligning with findings in *S. bicolor* [31], *Fragaria* [13], and *Brassica campestris* [32]. The isoelectric point (pI) ranges from 5.84 to 9.48, similar to the values observed in species such as *Solanum lycopersicum* [29], *Malus domestica* [33], *P. trichocarpa* [30], and *Medicago sativa* [25]. Predicted subcellular localization indicates that these proteins are primarily nuclear but may also function in the cytoplasm, chloroplasts, and periplasm, suggesting diverse roles across compartments. The aliphatic index ranges from 58.13 to 82.15, indicating that these proteins are likely to exhibit thermal stability, as reported in studies on *Fragaria* [13] and *Ipomoea batatas* [18].

Phylogenetic analysis using Neighbor-Joining (Figure 3) grouped ZAT genes into three subfamilies (ZAT-A, ZAT-B, ZAT-C). Notably, CqZAT5 and CqZAT6 (ZAT-B) clustered with genes from *A. thaliana* [20] and *Gossypium* [12] associated with salt stress tolerance, indicating potential roles in salinity tolerance in *C. quinoa*. The functionality of the sequences in the ZAT-A and ZAT-C groups of *C. quinoa* remains unclear. Although experimental studies in other species have linked these groups to general stress responses, it has not been confirmed whether the associated genes are functional in *C. quinoa* or if they are specifically related to salt tolerance.

The analysis of promoter regions (Figure 4) of the CqZAT genes reveals a rich variety of cis-elements, suggesting that these genes are involved in multiple physiological and biochemical activities in quinoa. The identification of elements associated with plant hormones, light, growth, and stress responses highlights the functional versatility of CqZAT genes as part of an adaptive response to challenging environments. The promoter regions of CqZAT4 and CqZAT8 contain TC-rich repeats, which are well-known elements in the promoters of stress-responsive genes, as demonstrated in studies on rice [34], maize [35], apple [36], and plum [37]. These studies have confirmed that such elements are commonly found in genes involved in abiotic stress responses, including salinity, drought, and cold.

### 3.2. Identification of a Halotolerant and a Halosensitive Accession in Chenopodium quinoa

The analysis of seed germination percentage under salt stress conditions was crucial for selecting a halotolerant accession (UNSA_VP033) and a halosensitive one (UNSA_VP021). UNSA_VP033 achieved a 99% survival rate at 500 mM and 64% at 700 mM. In contrast, UNSA_VP021 exhibited only a 4% survival rate at 500 mM, with no survival at 700 mM. The determination of the median lethal dose for *C. quinoa* plants was conducted under hydroponic system; for UNSA_VP021, it was 200 mM, whereas for UNSA_VP033, it was 300 mM.

The results show that the accession UNSA_VP033 exhibits greater tolerance to salt stress, as evidenced by increased dry matter and root length across all treatments, suggesting adaptations that sustain growth under adverse conditions. Additionally, it retains water more efficiently than UNSA_VP021, indicating superior capability in managing dehydration due to salinity. Regarding pigments, UNSA_VP033 significantly reduces its chlorophyll and carotenoid content under high salinity, likely as a mechanism to mitigate oxidative damage. This phenomenon is commonly observed in response to salt stress, where the accumulation of reactive oxygen species (ROS) can cause damage to cellular components, including lipids, proteins, and nucleic acids [38,39]. These responses are comparable to those seen in rice [40], cotton [41,42], and wheat [43,44,45], where salt-tolerant varieties exhibit superior management in conserving water in their tissues and improved root growth, enabling them to remain healthier and more productive under adverse conditions. Under high salinity, chlorophyll levels in plants decrease, as salt affects chloroplasts, inhibits photosynthesis, and reduces pigment biosynthesis. Salt-tolerant plants reduce chlorophyll and carotenoids to limit photosynthesis and ROS formation, protecting cells from damage. Furthermore, it is possible that *C. quinoa* maintains efficient ionic homeostasis, regulating sodium and potassium and redirecting resources towards defense mechanisms, such as antioxidants and osmoprotectants, without relying on increased pigment levels [46,47].

On the other hand, UNSA_VP021 shows a more limited response to salt stress. Although it increases its dry matter, root length only significantly increases at 200 mM, while its water retention capacity decreases, and chlorophyll and carotenoid content moderately increase under salinity at 100 mM and 200 mM. Salt-sensitive plants may temporarily elevate chlorophyll and carotenoid levels as an initial response to stress, attempting to compensate for damage and sustain photosynthesis. However, this increase can lead to long-term ROS accumulation, causing cellular damage and oxidative stress as salt stress persists [48]. While tolerant plants activate antioxidant signaling pathways to minimize damage, sensitive plants lack a robust response, exacerbating cellular damage [47,49]. Studies in species such as rice [50,51] and wheat [52] show variable responses: tolerant plants exhibit better control over pigment production to avoid oxidative damage, while sensitive plants fail to sustain photosynthesis effectively due to oxidative stress.

Previous studies support the results of this research on *Chenopodium quinoa’s* response to salt stress. Hussin et al. [48] and Razzaghi et al. [53] have documented that quinoa species exhibit significant adaptations to salinity, such as increased water retention capacity and enhanced root development compared to other species under salt stress.

### 3.3. Gene Expression Analysis in Tolerant and Sensitive Accessions of C. quinoa

**Figure A2:**
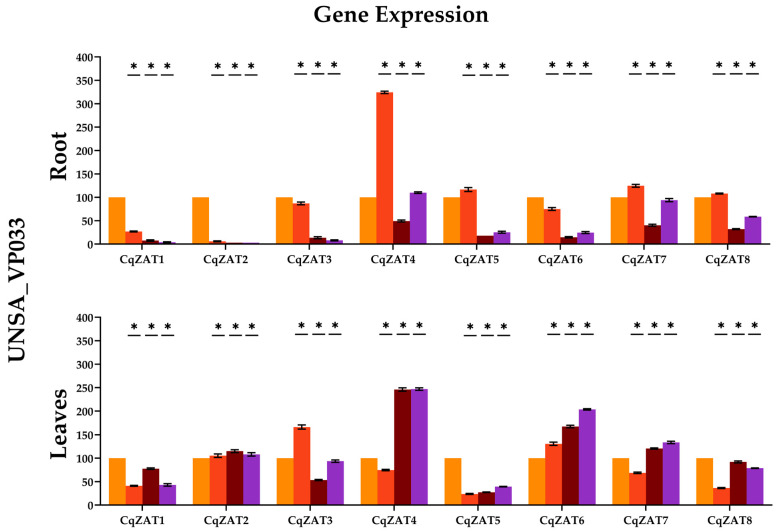
Relative Gene Expression in the Halotolerant Accession Under Different Concentrations Compared to the Control. The data were analyzed using ANOVA followed by Dunnett’s post-hoc test. Asterisks (*) indicate statistically significant differences between groups at a significance level of *p* < 0.05.

The results reveal variation in the relative expression levels of the CqZAT genes in response to salt stress, in both roots and leaves of the halotolerant and halosensitive accessions. In the root, it is observed that in the UNSA_VP033 accession, the genes show significant subexpression at 300 mM (Figure A2), whereas in UNSA_VP021, the expression is variable at 200 mM. In the leaves, it is observed that for UNSA_VP033 at 300 mM, the expression is variable, with CqZAT4 and CqZAT6 being overexpressed more than twofold compared to the control, whereas in UNSA_VP021 at 200 mM, the genes are underexpressed, with the exception of CqZAT4. In *C. quinoa*, studies by Panuccio et al. [54] and Hariadi et al. [55] indicate that the root plays a critical role in regulating osmotic and redox balance under salt stress conditions. This could suggest that the subexpression of the CqZAT genes in the UNSA_VP033 accession may be involved in these mechanisms. In the leaves, the overexpression of CqZAT4 and CqZAT6 in UNSA_VP033 could be key to the aerial part’s response to salt stress tolerance in quinoa, as observed in studies of salt-tolerant species such as *A. thaliana* [20] and *Gossypium* [12]. These studies analyze other genes from the ZAT family that differ in their amino acid sequences from CqZAT4 and CqZAT6, suggesting that this family could play an important role in the response to abiotic stress.

## 4. Materials and Methods

### 4.1. Identification of ZAT Genes, Members of the C1-2i Subclass in Chenopodium quinoa

To identify ZAT genes in *C. quinoa*, sequences of ZAT genes from *Arabidopsis thaliana* were downloaded from The Arabidopsis Information Resource (TAIR; http://www.arabidopsis.org, accessed on 22 March 2024). These Arabidopsis ZAT sequences were used as input for BLAST similarity searches on Phytozome v13 (https://phytozome-next.jgi.doe.gov/, accessed on 22 March 2024) and the National Center for Biotechnology Information (NCBI; https://www.ncbi.nlm.nih.gov/, accessed on 22 March 2024) to obtain genomic, coding, and protein sequences of ZAT genes in *C. quinoa.*

The obtained CqZAT sequences were submitted to InterProScan (https://www.ebi.ac.uk/interpro/search/sequence-search, accessed on 22 March 2024), CDD v3.19 (https://www.ncbi.nlm.nih.gov/Structure/bwrpsb/bwrpsb.cgi, accessed on 22 March 2024), and SMART v9.0 (http://smart.embl-heidelberg.de/, accessed on 22 March 2024) to confirm the presence of the conserved C2H2 domain. Redundant sequences were manually removed.

Physicochemical properties of CqZAT proteins, including sequence length, molecular weight, isoelectric point, and aliphatic index, were predicted using the ExPASy ProtParam web tool (https://web.expasy.org/protparam/, accessed on 23 March 2024). Subcellular localization predictions were performed using the web tools GENSCRIPT (https://www.genscript.com/wolf-psort.html, accessed on 23 March 2024), CELLO (http://cello.life.nctu.edu.tw, accessed on 23 March 2024), Plant-mPLoc (www.csbio.sjtu.edu.cn/bioinf/plant-multi/, accessed on 23 March 2024), and WoLF PSORT (http://wolfpsort.hgc.jp, accessed on 23 March 2024).

### 4.2. Domain Evaluation, Gene Structure Analysis, and Prediction of Conserved Motifs

The presence and positions of zinc finger domains in *C. quinoa* ZAT proteins were identified using data from CDD v3.19 (https://www.ncbi.nlm.nih.gov/Structure/bwrpsb/bwrpsb.cgi, accessed on 24 March 2024). Gene structure analysis was performed using a GFF annotation file downloaded from the Phytozome v13 quinoa database (https://phytozome-next.jgi.doe.gov/, accessed on 24 March 2024).

Conserved motif analysis was conducted using Multiple Em for Motif Elicitation (MEME) version 5.1.1 (https://meme-suite.org/meme/tools/meme, accessed on 24 March 2024) with the following parameters: unlimited repeat occurrences, a maximum of 10 motifs, and an optimal motif width of 4–50 residues. Graphical outputs were visualized using TBtools v2.019.

### 4.3. Sequence Alignment and Phylogenetic Analysis

A multiple sequence alignment of *C. quinoa* proteins was performed using Clustal Omega with default parameters. The results were visualized using JALVIEW. These aligned sequences were subsequently used to conduct phylogenetic analysis with Molecular Evolutionary Genetic Analysis (MEGA v.11).

Another multiple sequence alignment was performed, this time including four additional species: *Arabidopsis thaliana*, *Gossypium hirsutum*, *Oryza sativa*, and *Sorghum bicolor*. For this, a sequence search was conducted for members of the ZAT gene family in the mentioned species, referencing 20 ZAT family members from *Arabidopsis*. Protein sequences for *O. sativa* were downloaded from Phytozome (https://phytozome.jgi.doe.gov/pz/portal.html, accessed 26 March 2024), genome sequences for *G. hirsutum* were obtained from the Gossypium Resource and Network Database (http://grand.cricaas.com.cn/home, accessed 26 March 2024), and genome sequences for *Arabidopsis* were retrieved from TAIR 10 (http://www.arabidopsis.org, accessed 26 March 2024). The four sequences were aligned using Clustal Omega with default parameters, and a phylogenetic tree was constructed employing the Neighbor-Joining algorithm and the Jones-Taylor-Thornton (JTT) substitution model with gamma distribution (G). The analysis included bootstrap resampling with 1000 replicates and a 50% cutoff threshold. The resulting phylogenetic tree data were exported as a Newick file and subsequently imported into iTOL (https://itol.embl.de/, accessed 28 March 2024) for further modification.

### 4.4. Analysis of Cis-Acting Promoter Elements in CqZAT Genes

Promoter sequences (2000 bp upstream of CqZAT coding sequences) were analyzed using the PlantCARE database (https://bioinformatics.psb.ugent.be/webtools/plantcare/html/, accessed on 25 March 2024) to predict cis-acting elements. Results were visualized with TBtools v2.019.

### 4.5. Plant Material and Experimental Treatments

A total of 36 accessions from the Germplasm Bank of the Genetics Laboratory at the National University of San Agustín were used, originating from various locations in the Altiplano region of the country.

a. Germination Percentage

To determine the germination percentages, the procedure described by Alanoca et al. (2013) [56] was followed with modifications, utilizing the paper method in Petri dishes.

A total of 100 seeds from each quinoa accession were counted and pre-disinfected with 2% sodium hypochlorite for 5 min, followed by thorough rinsing with distilled water to prevent contamination. The seeds were placed on moistened filter paper with sterilized distilled water, arranged in three replicates of 100 seeds each, and properly labeled with the sowing date.

Subsequently, the seeds were incubated in a germination chamber at a temperature of 22 °C, with a photoperiod of 11 h of light and 13 h of darkness. Germination percentages were calculated using the following formula [57]:

Germination Percentage = (Number of Germinated Seeds)/(Total Seeds) × 100%(1)

b. Selection of Halotolerant and Halosensitive Accessions

Accessions with a germination percentage greater than 90% were selected for evaluation. These accessions were subjected to eight treatments with different NaCl concentrations: 0 mM, 100 mM, 200 mM, 300 mM, 400 mM, 500 mM, 600 mM, and 700 mM. These different concentrations were used to evaluate the response of *C. quinoa* accessions to salinity stress, allowing us to identify tolerant accessions, which are those capable of germinating and growing at high salinity levels, and sensitive ones, which exhibit reduced performance at higher concentrations. Each treatment had three replicates.

Seeds were sterilized by immersion in 2% sodium hypochlorite for 5 min and then rinsed thoroughly with distilled water. Sterilized seeds were placed in Petri dishes containing a sheet of paper and 5 mL of the respective treatment solution. Each dish was labeled with the accession code, NaCl concentration, and date. Evaluations were performed five days after sowing [58].

c. *Chenopodium quinoa* Seedlings Subjected to Saline Stress in Hydroponic Systems

Accessions exhibiting the highest and lowest tolerance to saline stress were selected for this phase of the study.

Seeds were sterilized with 2% sodium hypochlorite and germinated on moistened filter paper for 4 days at 21 °C. Following the modified methodology of Cole et al. (2020) [59], the seedlings were transferred to hydroponic containers with Hoagland nutrient solution at a 1X concentration (Table A3). The growth chamber was maintained at 24 °C, with a photoperiod of 16 h and humidity levels of 50–70%. After 24 days, when the seedlings developed their third true leaf, they were subjected to saline treatments with sodium chloride.

To evaluate the response of halotolerant and halosensitive accessions to salinity stress, different salt treatments were applied at concentrations of 0 mM, 100 mM, 200 mM, 300 mM, and 400 mM to determine their median lethal dose. Accordingly, the salt-tolerant accession was subjected to four salinity treatments: 0 mM, 100 mM, 200 mM, and 300 mM; while the salt-sensitive accession was subjected to three treatments: 0 mM, 100 mM, and 200 mM. Each treatment was performed in triplicate, and after a period of 5 days, leaves and roots were collected from three individual seedlings per biological replicate. These samples were immediately placed in liquid nitrogen and stored at −80 °C.

d. Determination of Morphological and Physiological Parameters
  - Dry Matter and Plant Length: Dry matter percentage was determined using the formula:
    Dry Matter (%) = (Dry Sample Weight)/(Fresh Sample Weight) × 100(2)
    Following the methodology of Ulloa and Valle (2021) [60], five samples were taken from each biological replicate for each treatment. The fresh weight of both the aerial and root parts was measured using a precision balance. The samples were then oven-dried at 80 °C for 24 h to determine their dry weight. Post-drying, the samples were weighed to calculate the dry mass of aerial parts (leaves + stems) and roots.
    The length of the aerial part of each sample was measured using graph paper, recording the measurements in millimeters. Root length was similarly measured from the base of the plant to the tip of the longest root. Fresh weight, dry weight, and length data were recorded for subsequent analysis.
  - Relative Water Content (RWC): RWC was determined following the methodology of Jensen et al. (2000) [61]. For each treatment, five leaves were collected from the middle third of 10 different plants, and their fresh weight (FW) was recorded immediately to avoid water loss due to transpiration. The leaves were then immersed in distilled water for 24 h at 24 °C under low light conditions. Afterward, the turgid weight (TW) was recorded, and leaves were oven-dried at 80 °C for 24 h to measure the dry weight (DW). The RWC was calculated using the following formula:
    RWC (%) = 100 × ((FW − DW)/(TW − DW))(3)
  - Chlorophyll Content: Chlorophyll content was determined according to the methodology of Sumanta et al. (2014) [62]. Precisely 0.5 g of fresh leaf tissue was weighed and ground in a mortar with 10 mL of cold 95% ethanol. The mixture was centrifuged at 10,000 rpm for 15 min, and the supernatant was collected. A 0.5 mL aliquot of the supernatant was mixed with 4.5 mL of cold 95% ethanol. Chlorophyll-a, chlorophyll-b, and total carotenoid contents were quantified using an Epoch2 microplate spectrophotometer (BioTek, New Haven, CT, USA) at absorbances of 664 nm, 649 nm, and 470 nm, respectively. The equations used for quantification are as follows:

Chl_a_ = 13.36A_664_ − 5.19A_649_ = mg/L (4)

Chl_b_ = 27.43A_649_ − 8.12A_664_ = mg/L (5)

Chl_T_ = 5.25A_664_ + 22.24A_649_ = mg/L (6)

C_T_ = (1000A_470_ − 2.13Chl_a_ − 97.63Chl_b_)/209 = mg/L (7)

where Chl_a_, Chl_b_, and Chl_T_ represent chlorophyll-a, chlorophyll-b, and total chlorophyll content, respectively, and C_T_ denotes total carotenoid content [63].

e. Statistical Analysis

The statistical analysis was structured to assess intergroup differences employing two primary methodologies. For pairwise comparisons of means between two independent groups, Student’s *t*-test was utilized under the assumptions of data normality and homogeneity of variances. Statistical significance was defined at a threshold of *p* < 0.05.

For comparisons involving more than two groups, a one-way analysis of variance (ANOVA) was performed to detect global differences across group means. In instances where the two-way ANOVA revealed statistical significance (*p* < 0.05), Dunnett’s post-hoc test was conducted to perform targeted comparisons between the control group and experimental groups. All statistical analyses were executed using SPSS Statistics (version 30.0.0).

### 4.6. Primer Design

Primers were designed based on the obtained CqZAT sequences (Table A4). These primers were designed to have a melting temperature (Tm) between 58 °C and 61 °C, a length of 18 to 25 base pairs, and an amplicon length of 100 to 200 bp, using Primer3Plus (https://www.bioinformatics.nl/cgi-bin/primer3plus/primer3plus.cgi, accessed 30 March 2024).

### 4.7. RNA Extraction and qPCR Analysis

RNA extraction was performed using 100 mg of root tissue and 90 mg of leaf tissue. The plant material was ground into a fine powder in liquid nitrogen using a mortar and pestle. The Spectrum Plant Total RNA Kit (Sigma-Aldrich-Merck, Darmstadt, Germany) was used for RNA extraction according to the manufacturer’s instructions. RNA concentration and purity were measured using a Biotech Epoch 2 spectrophotometer, and the RNA was stored at −80 °C until use.

The SYBR Green GoTaq^®^ 2-Step RT-qPCR system (Promega, Madison, WI, USA) was used for real-time quantitative PCR (qRT-PCR) in a two-step protocol. The first step involved cDNA synthesis, followed by the qPCR protocol, as per the manufacturer’s specifications.

The protocol for cDNA synthesis begins with the denaturation of RNA (5 µg) combined with Oligo (dT)15 primers and Random Primers (1 µg each), with nuclease-free water added to a final volume of 10 µL. After thermal treatment at 70 °C, a reverse transcription mix is prepared consisting of 1.5 µL nuclease-free water, 4 µL buffer, 2 µL MgCl_2_, 1 µL nucleotides, 0.5 µL ribonuclease inhibitor, and 1 µL reverse transcriptase, making a total of 10 µL. This mixture is added to the denatured RNA, resulting in a final reaction volume of 20 µL. The reaction is incubated at 25 °C and then at 42 °C for 1 h. The reverse transcriptase is inactivated by heating to 70 °C for 15 min, and the cDNA is stored at 4 °C for short-term use or −20 °C for long-term storage.

For the qPCR protocol, a reaction mixture is prepared consisting of 10 µL GoTaq^®^ qPCR Master Mix, 1 µL Forward Primer, 1 µL Reverse Primer, 4 µL cDNA, and nuclease-free water to reach a total volume of 20 µL. For internal control, the reference gene Glyceraldehyde-3-phosphate dehydrogenase b (GAPDH, GenBank ID: XM_021862063.1) was used. The reaction mix is prepared at room temperature, distributed into PCR tubes or wells, sealed, centrifuged, and subjected to thermal cycling.

The thermal cycling conditions were as follows: 95 °C for 2 min, followed by 40 cycles of 95 °C for 15 s and 61 °C for 1 min. Each biological replicate was analyzed with three technical replicates. Gene expression levels of the CqZAT genes were calculated using the 2^−∆∆CT^ method.

## 5. Conclusions

This study demonstrates that ZAT genes, particularly CqZAT4 and CqZAT6, play a key role in the salinity stress response of *Chenopodium quinoa*. Their higher expression in the tolerant accession (UNSA_VP033) under saline conditions suggests their involvement in regulating ionic homeostasis and antioxidant defenses. Furthermore, the identification of cis-regulatory elements in their promoter regions reinforces their role in the physiological and biochemical adaptation to adverse conditions.

These findings provide valuable insights for the genetic improvement of quinoa, enabling the development of more salt-tolerant varieties. Future studies should focus on the functional evaluation of the identified genes to elucidate their mechanisms of action and enhance their application in agricultural strategies to combat abiotic stress, positioning quinoa as a strategic resource for global food security.

## Figures and Tables

**Table 1:** Physicochemical properties of ZAT proteins from the C2H2 zinc finger family, subclass C1-2i, in *Chenopodium quinoa*.

Phylogenetic Group	Gen	Gene Report	Location	Gene (pb *)	CDS * (pb *)	PP * (aa *)	MW * (kDa *)	Aliphatic Index	pI *	Cellular Location *
ZAT-A	CqZAT1	AUR62001834	C_Quinoa_Scaffold_2716:9075033..9075780 reverse	747	747	248	26.32	67.66	6.66	Nu ^1,3,4^, Cy ^1^, Ec ^2^, Pe ^2^
ZAT-C	CqZAT2	AUR62038382	C_Quinoa_Scaffold_1034:1966184..1966709 reverse	525	525	174	19.38	72.30	9.48	Nu ^1,2,3,4^, Cp ^1^, Ec ^2^
ZAT-C	CqZAT3	AUR62038383	C_Quinoa_Scaffold_2493:1671380..1672130 reverse	614	534	177	19.71	82.15	9.10	Nu ^1,3,4^, Cy ^2^, Ec ^2^
ZAT-C	CqZAT4	AUR62039327	C_Quinoa_Scaffold_1932:1438123..1440062 reverse	1939	1068	355	38.12	64.39	5.84	Nu ^1,2,3,4^
ZAT-B	CqZAT5	XP_021725368	NW_018744124.1 (330074..331406)	1333	717	238	25.58	61.55	8.79	Nu ^1,2,3,4^
ZAT-B	CqZAT6	XP_021728442	NW_018744301.1 (4034356..4035720)	1365	789	262	28.14	58.13	9.13	Nu ^1,2,3,4^
ZAT-C	CqZAT7	XP_021766394	NW_018743050.1 (1137896..1139053, complement)	1158	1005	334	35.34	64.34	5.94	Nu ^1,3,4^, Cy ^1^, Ec ^2^
ZAT-C	CqZAT8	XP_021774557	NW_018743389.1 (4219259..4221239, complement)	1981	990	329	35.79	60.52	6.69	Nu ^1,2,3,4^

* Abbreviations: CDS, Coding DNA Sequence; PP, Polypeptide; MW, Molecular Weight; pI, Isoelectric Point; bp, Base Pair; aa, Amino Acid; kDa, Kilodalton; Ec, Extracellular; Cp, Chloroplast; Cy, Cytoplasm; Pe, Periplasm; Nu, Nucleus. ^1^ https://www.genscript.com/wolf-psort.html (accessed on 23 March 2024); ^2^ http://cello.life.nctu.edu.tw (accessed on 23 March 2024); ^3^ www.csbio.sjtu.edu.cn/bioinf/plant-multi/ (accessed on 23 March 2024); ^4^ http://wolfpsort.hgc.jp (accessed on 23 March 2024)

## Data Availability

The data presented in this study are available upon request from the corresponding author.

## Supplementary Materials

The following supporting information can be downloaded at: https://www.mdpi.com/article/10.3390/ijms26062570/s1.

## Abbreviations

The following abbreviations are used in this manuscript:

MDPI	Multidisciplinary Digital Publishing Institute
DOAJ	Directory of open access journals
TLA	Three letter acronym
LD	Linear dichroism

## Appendix A

**Table A1:** Frequency of amino acids present in the CqZATs sequences.

Abbreviation	Name	Freq,
**A**	Alanine	0.0808
**C**	Cysteine	0.0246
**D**	Aspartic acid	0.0477
**E**	Glutamic acid	0.0567
**F**	Phenylalanine	0.0269
**G**	Glycine	0.0704
**H**	Histidine	0.043
**I**	Isoleucine	0.0373
**K**	Lysine	0.0614
**L**	Leucine	0.0737
**M**	Methionine	0.026
**N**	Asparagine	0.0633
**P**	Proline	0.0435
**Q**	Glutamine	0.0288
**R**	Arginine	0.0463
**S**	Serine	0.121
**T**	Threonine	0.0831
**V**	Valine	0.0477
**W**	Tryptophan	0.00236
**Y**	Tyrosine	0.0156

**Table A2:** Motif, sequence and function of the cis-elements presented in Figure 2.

Cis-Element	Sequence	Function
***Plant Hormone***		
**ABRE**	ACGTG	cis-acting element involved in the abscisic acid responsiveness
**TGACG-motif**	TGACG	cis-acting regulatory element involved in the MeJA-responsiveness
**CGTCA-motif**	CGTCA	cis-acting regulatory element involved in the MeJA-responsiveness
**TATC-box**	TATCCCA	cis-acting element involved in gibberellin-responsiveness
**TCA-element**	CCATCTTTTT	cis-acting element involved in salicylic acid responsiveness
***Stress Responsiveness***		
**GC-motif**	CCCCCG	enhancer-like element involved in anoxic specific inducibility
**LTR**	CCGAAA	cis-acting element involved in low-temperature responsiveness
**MBS**	CAACTG	MYB binding site involved in drought-inducibility
**TC-rich repeats**	GTTTTCTTAC	cis-acting element involved in defense and stress responsiveness
**WUN-motif**	AAATTTCCT	wound-responsive element
***Plant Growth***		
**AACA_motif**	TAACAAACTCCA	involved in endosperm-specific negative expression
**ARE**	AAACCA	cis-acting regulatory element essential for the anaerobic induction
**CAT-box**	GCCACT	cis-acting regulatory element related to meristem expression
**circadian**	CAAAGATATC	cis-acting regulatory element involved in circadian control
**GCN4_motif**	TGAGTCA	cis-regulatory element involved in endosperm expression
**MBSI**	aaaAaaC(G/C)GTTA	MYB binding site involved in flavonoid biosynthetic genes regulation
**O2-site**	GATGATGTGG	cis-acting regulatory element involved in zein metabolism regulation
***Light Responsiveness***		
**G-Box**	CACGTT	cis-acting regulatory element involved in light responsiveness
**AAAC-motif**	CAATCAAAACCT	light responsive element
**GT1-motif**	GGTTAAT	light responsive element
**Sp1**	GGGCGG	light responsive element
**MRE**	AACCTAA	MYB binding site involved in light responsiveness
**ATC-motif**	AGTAATCT	part of a conserved DNA module involved in light responsiveness
**ATCT-motif**	AATCTAATCC	part of a conserved DNA module involved in light responsiveness
**Box 4**	ATTAAT	part of a conserved DNA module involved in light responsiveness
**chs-CMA1a**	TTACTTAA	part of a light responsive element
**GA-motif**	ATAGATAA	part of a light responsive element
**Gap-box**	CAAATGAA(A/G)A	part of a light responsive element
**GATA-motif**	AAGATAAGATT	part of a light responsive element
**I-box**	AGATAAGG	part of a light responsive element
**TCCC-motif**	TCTCCCT	part of a light responsive element
**TCT-motif**	TCTTAC	part of a light responsive element
**AE-box**	AGAAACAA	part of a module for light response

**Table A3:** Mineral nutrient solutions according to Hoagland.

Symbol	Compound	Concentration	
		Molarity	g/L
**A**	Ca(NO_3_)_2_.4H_2_O	1.00 M	236.00
**B**	KNO_3_	1.00 M	101.00
**C**	MgSO_4_.7H_2_O	1.00 M	247.00
**D**	KH_2_PO_4_	1.00M	136.00
**E**	Micronutrientes		
**F**	Fe-EDTA		
**Micronutrients**	**Concentration g/L**	**Micronutrients**	**Concentration g/L**
**MnCl_2_.4H_2_O**	1.81	H_3_BO_3_	2.86
**ZnSO_4_.7H_2_O**	0.22	CuSO_4_.5H_2_O	0.10
**H_2_MoO_4_.H_2_O**	0.10		

**Table A4:** Primers Used for CqZAT Sequences.

Code	Gen		Primers
**AUR62001834**	CqZAT1	>CqZAT1_F	GTTGGAGGTGGAAGTTCTGAG
		>CqZAT1_R	TTACACGAGTGAGCCTTCGG
**AUR62038382**	CqZAT2	>CqZAT2_F	TAAAGACCGCAACCGTGACA
		>CqZAT2_R	CGGTTTTCCCTTCGTTGTATG
**AUR62038383**	CqZAT3	>CqZAT3_F	AGCGACGTAAGAAAGGCGAAA
		>CqZAT3_R	TAAGCGAAAGCGAAAGCGAAAG
**AUR62039327**	CqZAT4	>CqZAT4_F	TCGTCAGATGATGAAGAACCAC
		>CqZAT4_R	TGCTCGATTTGTCAGTTGAAGT
**XP_021725368**	CqZAT5	>CqZAT5_F	GTGGAGTATGTGACAAGGAGTTTCT
		>CqZAT5_R	CAGTGGTTGCTATTGTGGTATGTAA
**XP_021728442**	CqZAT6	>CqZAT6_F	TGATTCCCGTCTATCCGAAG
		>CqZAT6_R	TATTGCTCTGCTTGGTGTCG
**XP_021766394**	CqZAT7	>CqZAT7_F	CCGAGTTTCCGTCAGGACAA
		>CqZAT7_R	GCAGTGGTGGTACTTATTGGAGA
**XP_021774557**	CqZAT8	>CqZAT8_F	AAAGCGAAGGTGCACGAATG
		>CqZAT8_R	CGCTTCGTGATGGACTAGGG

**Table A5:** Salinity Response at Different Concentrations in Chenopodium quinoa Accessions.

Accession	Repetition	0 mM	100 mM	200 mM	300 mM	400 mM	500 mM	600 mM	700 mM
**UNSA_VP002**	1	90	84	60	46	30	24	0	0
	2	95	84	64	47	32	26	0	0
	3	92	84	62	44	31	25	0	0
**UNSA_VP003**	1	91	86	62	46	33	23	0	0
	2	93	83	60	43	30	24	0	0
	3	90	85	63	45	32	26	0	0
**UNSA_VP004**	1	94	82	58	45	29	22	0	0
	2	91	81	60	48	30	23	0	0
	3	89	83	61	43	31	24	0	0
**UNSA_VP010**	1	93	92	86	64	32	19	0	0
	2	90	90	84	65	29	19	0	0
	3	94	94	83	66	30	21	0	0
**UNSA_VP011**	1	92	96	93	90	88	30	0	0
	2	97	95	93	90	86	28	0	0
	3	99	97	95	91	85	31	0	0
**UNSA_VP014**	1	91	84	80	78	82	14	0	0
	2	90	80	80	78	91	17	0	0
	3	91	82	79	77	80	13	0	0
**UNSA_VP015**	1	90	88	74	70	50	36	0	0
	2	95	85	78	72	56	37	0	0
	3	97	81	89	72	52	34	0	0
**UNSA_VP016**	1	88	68	78	54	36	18	0	0
	2	85	68	78	54	30	19	0	0
	3	87	67	77	52	35	15	0	0
**UNSA_VP017**	1	100	100	100	100	100	72	40	2
	2	100	100	100	100	96	56	34	5
	3	100	100	100	100	92	50	35	4
**UNSA_VP018**	1	95	90	88	70	91	81	0	0
	2	91	92	94	76	92	80	0	0
	3	93	94	96	71	94	82	0	0
**UNSA_VP019**	1	90	100	94	84	32	20	0	0
	2	91	99	93	83	31	21	0	0
	3	92	100	95	85	33	17	0	0
**UNSA_VP020**	1	100	100	100	100	98	70	59	36
	2	99	99	99	99	97	68	40	33
	3	100	100	100	100	98	72	45	32
**UNSA_VP021**	1	100	95	97	39	22	5	0	0
	2	100	97	95	41	25	3	0	0
	3	100	94	97	42	23	5	0	0
**UNSA_VP022**	1	100	94	98	98	29	16	5	2
	2	94	94	100	96	28	14	8	4
	3	100	100	100	97	35	15	6	2
**UNSA_VP028**	1	92	90	74	58	36	20	0	0
	2	95	89	75	57	35	19	0	0
	3	93	91	78	59	38	21	0	0
**UNSA_VP030**	1	97	92	93	92	90	19	0	0
	2	96	91	93	91	92	20	0	0
	3	98	93	95	93	94	23	0	0
**UNSA_VP033**	1	100	100	99	100	98	100	78	69
	2	98	96	98	100	96	98	80	65
	3	100	99	98	100	96	100	77	57
**UNSA_VP036**	1	96	94	96	90	56	33	0	0
	2	100	96	94	70	54	30	0	0
	3	98	92	96	86	44	32	0	0

**Table A6:** Percentage survival of Halotolerant and a Halosensitive Accession in *Chenopodium quinoa* in a hydroponic system.

Accession	Repetition	Concentration					
		0 mM	100 mM	200 mM	300 mM	400 mM	500 mM
**UNSA_VP021**	1	100	100	100	100	10	0
	2	100	100	100	100	11	0
	3	100	100	100	100	10	0
**UNSA_VP033**	1	100	100	100	32	0	0
	2	100	100	100	34	0	0
	3	100	100	100	36	0	0

**Table A7:** Salinity Response at Different Concentrations in a Halotolerant and a Halosensitive Accession of *Chenopodium quinoa* Based on Dry Matter Percentage (%).

Accession	Repet	Control		100 mM		200 mM		300 mM	
		Leave	Root	Leave	Root	Leave	Root	Leave	Root
**UNSA_VP021**	**1**	8.29	8.55	9.15	9.10	11.14	10.32	-	-
	**2**	8.72	6.92	8.97	8.08	10.98	10.08	-	-
	**3**	7.67	7.30	8.99	9.08	11.02	10.41	-	-
**UNSA_VP031**	**1**	8.72	10.92	10.68	8.82	10.56	10.69	11.48	10.46
	**2**	8.68	10.80	9.96	8.62	10.73	10.80	12.03	10.41
	**3**	7.98	11.22	9.42	9.19	10.57	10.70	11.68	10.42

**Table A8:** Salinity Response at Different Concentrations in a Halotolerant and a Halosensitive Accession of *Chenopodium quinoa* Based on Root Length (mm).

Accession	Repetition	Control	100 mM	200 mM	300 mM
**UNSA_VP021**	**1**	261.4	234.4	254	-
	**2**	213.6	275.6	274	-
	**3**	248	238	266	-
**UNSA_VP031**	**1**	146	217.4	241.8	234.6
	**2**	193.6	179	183.8	228.6
	**3**	157.4	223.2	196.1	242

**Table A9:** Salinity Response at Different Concentrations in a Halotolerant and a Halosensitive Accession of *Chenopodium quinoa* Based on Relative Water Content (%).

Accession	Repetition	Control	100 mM	200 mM	300 mM
**UNSA_VP021**	**1**	69.55	59.05	47.66	-
	**2**	73.87	55.73	47.22	-
	**3**	74	58	47	-
**UNSA_VP031**	**1**	79.71	65.71	57.10	64.93
	**2**	79.44	68.48	59.62	65.08
	**3**	76.87	64.46	58.28	65.32

**Table A10:** Salinity Response at Different Concentrations in a Halotolerant and a Halosensitive Accession of *Chenopodium quinoa* Based on Chlorophyll A, Chlorophyll B, Total Chlorophyll, and Total Carotenoid Content (µg/mL).

			UNSA_VP021			UNSA_VP033			
		Repetition	Control	100 mM	200 mM	Control	100 mM	200 mM	300 mM
**Chlorophyll**	**A**	**1**	2.33	2.58	2.61	2.63	2.59	2.57	2.00
		**2**	2.51	2.52	2.77	2.51	2.05	2.48	2.08
		**3**	2.71	2.86	2.89	3.01	2.47	2.21	2.35
	**B**	**1**	0.77	0.89	0.79	0.84	0.89	0.91	0.66
		**2**	0.81	0.83	0.86	0.79	0.67	0.82	0.67
		**3**	0.87	0.98	0.89	0.96	0.84	0.70	0.74
	**Total**	**1**	29.25	33.27	31.26	32.38	33.30	33.64	25.11
		**2**	31.13	31.76	33.68	30.84	25.70	31.14	25.85
		**3**	33.45	36.85	34.87	37.17	31.72	27.06	28.83
**Total Carotens**		**1**	1.23	1.40	1.37	1.41	1.44	1.44	1.11
		**2**	1.32	1.34	1.45	1.35	1.15	1.35	1.16
		**3**	1.44	1.49	1.51	1.61	1.39	1.20	1.24

**Table A11:** Salinity Response at Different Concentrations in a Halosensitive Accession (UNSA_VP021) of *Chenopodium quinoa* Analyzing CqZAT Gene Expression Levels (%).

Organ	Treat	Repetition	CqZAT1	CqZAT2	CqZAT3	CqZAT4	CqZAT5	CqZAT6	CqZAT7	CqZAT8
**Roots**	**0 mM**	**1**	100.0	100.0	100.0	100.0	100.0	100.0	100.0	100.0
		**2**	100.0	100.0	100.0	100.0	100.0	100.0	100.0	100.0
		**3**	100.0	100.0	100.0	100.0	100.0	100.0	100.0	100.0
	**100 mM**	**1**	76.4	127.8	29.6	76.2	37.5	62.8	125.5	107.5
		**2**	78.6	122.5	30.0	75.9	36.8	65.4	125.8	109.0
		**3**	79.1	123.0	27.6	79.3	35.4	67.8	124.7	110.8
	**200 mM**	**1**	31.49	100.20	18.64	117.01	30.52	67.00	159.00	103.13
		**2**	26.84	101.38	18.41	112.50	29.95	70.95	160.88	101.26
		**3**	24.47	99.32	18.11	116.72	30.72	69.52	157.23	103.87
**Leaves**	**0 mM**	**1**	100.0	100.0	100.0	100.0	100.0	100.0	100.0	100.0
		**2**	100.0	100.0	100.0	100.0	100.0	100.0	100.0	100.0
		**3**	100.0	100.0	100.0	100.0	100.0	100.0	100.0	100.0
	**100 mM**	**1**	18.28	36.18	17.76	332.44	7.44	6.68	62.21	138.13
		**2**	16.24	28.78	13.35	335.34	7.21	4.19	53.99	139.35
		**3**	19.68	29.19	11.69	339.64	6.83	4.10	56.29	133.70
	**200 mM**	**1**	11.94	23.46	28.76	203.16	21.45	7.60	55.84	100.61
		**2**	11.40	26.58	23.72	204.56	17.48	8.40	48.70	103.00
		**3**	12.34	29.08	24.81	207.27	20.87	7.49	48.13	101.91

**Table A12:** Salinity Response at Different Concentrations in a Halotolerant Accession (UNSA_VP033) of *Chenopodium quinoa* Analyzing CqZAT Gene Expression Levels (%).

Organ	Treat.	Repetition	CqZAT1	CqZAT2	CqZAT3	CqZAT4	CqZAT5	CqZAT6	CqZAT7	CqZAT8
**Roots**	**0 mM**	**1**	100.0	100.0	100.0	100.0	100.0	100.0	100.0	100.0
		**2**	100.0	100.0	100.0	100.0	100.0	100.0	100.0	100.0
		**3**	100.0	100.0	100.0	100.0	100.0	100.0	100.0	100.0
	**100 mM**	**1**	28.06	6.79	89.57	323.68	119.83	75.40	128.43	109.30
		**2**	26.50	6.16	87.29	321.65	118.08	78.36	121.78	106.51
		**3**	26.76	4.75	84.42	326.98	112.49	72.16	124.02	107.96
	**200 mM**	**1**	5.52	3.07	12.10	49.79	17.63	14.52	41.35	32.40
		**2**	7.59	3.10	13.03	51.06	17.91	12.86	42.03	31.05
		**3**	8.58	3.14	15.53	47.40	17.55	16.02	37.56	32.82
	**300 mM**	**1**	3.31	2.53	7.58	107.58	25.92	27.32	90.15	57.53
		**2**	4.98	3.49	7.49	110.64	23.22	24.08	95.51	59.27
		**3**	4.33	3.07	8.77	110.84	27.30	23.25	95.53	59.42
**Leaves**	**0 mM**	**1**	100.0	100.0	100.0	100.0	100.0	100.0	100.0	100.0
		**2**	100.0	100.0	100.0	100.0	100.0	100.0	100.0	100.0
		**3**	100.0	100.0	100.0	100.0	100.0	100.0	100.0	100.0
	**100 mM**	**1**	41.17	107.05	161.22	73.15	24.56	134.03	69.56	37.09
		**2**	39.50	107.80	170.03	76.03	22.59	127.35	66.99	34.56
		**3**	41.69	101.28	167.20	74.79	22.51	130.55	68.55	36.66
	**200 mM**	**1**	78.73	118.37	51.92	249.03	26.93	164.66	122.46	94.37
		**2**	76.32	115.14	53.60	246.66	27.49	169.63	120.19	90.15
		**3**	77.77	111.60	53.79	241.64	28.32	165.64	119.70	91.60
	**300 mM**	**1**	40.83	107.05	91.48	250.32	39.41	205.18	130.86	78.79
		**2**	42.44	111.60	96.31	246.28	40.04	202.34	134.29	77.52
		**3**	45.77	104.85	94.11	244.97	38.54	203.66	136.48	79.15
