# Supplementary figures and images for: In Silico Characterization and Determination of Gene Expression Levels Under Saline Stress Conditions in the Zinc Finger Family of the C1-2i Subclass in Chenopodium quinoa Willd
Source: Int J Mol Sci. 2025 Mar 13;26(6):2570. doi: 10.3390/ijms26062570 (PMC11942331; doi:10.3390/ijms26062570)

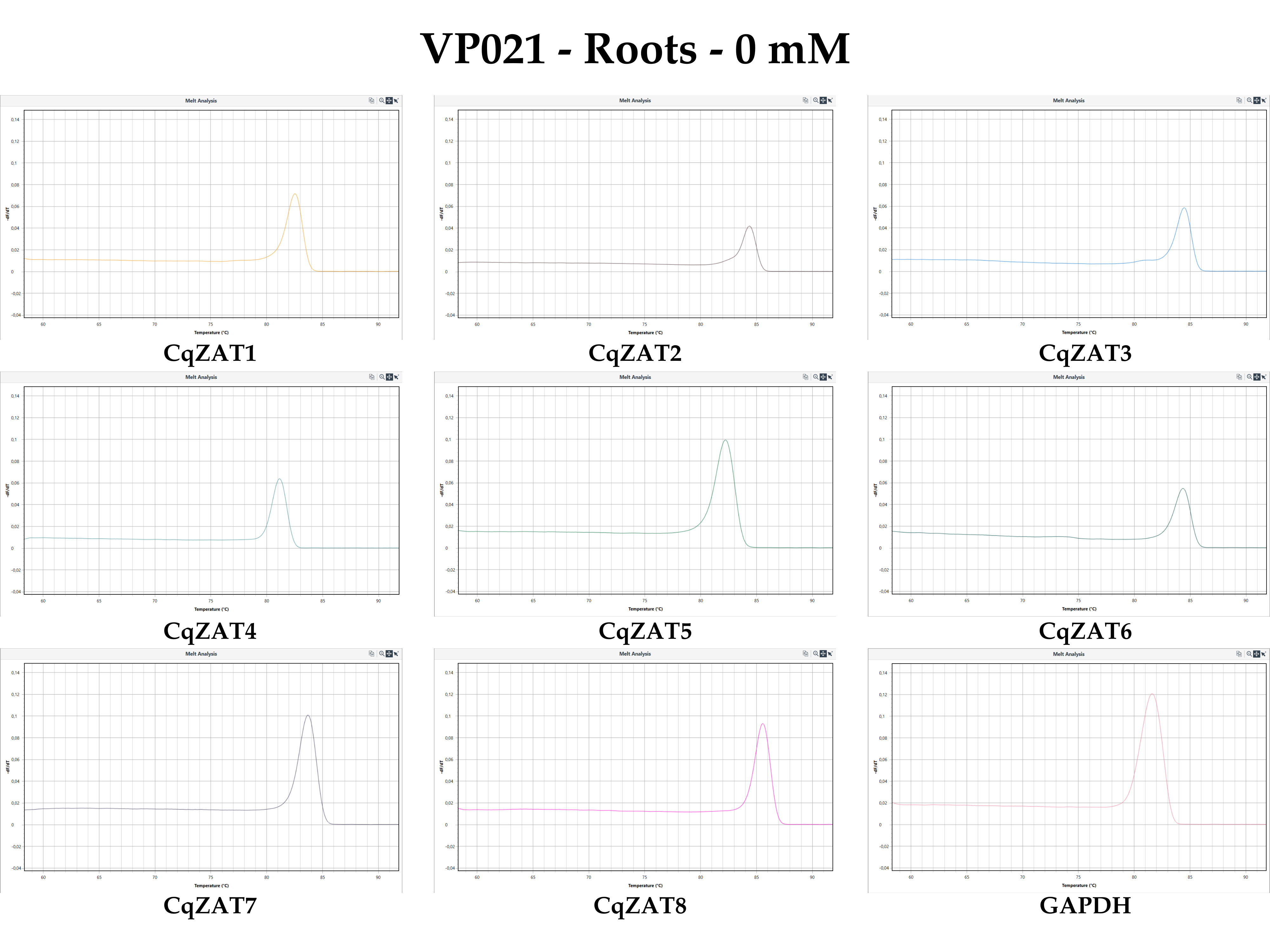

Supplement: Supplementary file 1 [file ijms-26-02570-s001.zip › Figure S1 - VP021 - Root - 0 mM.png]

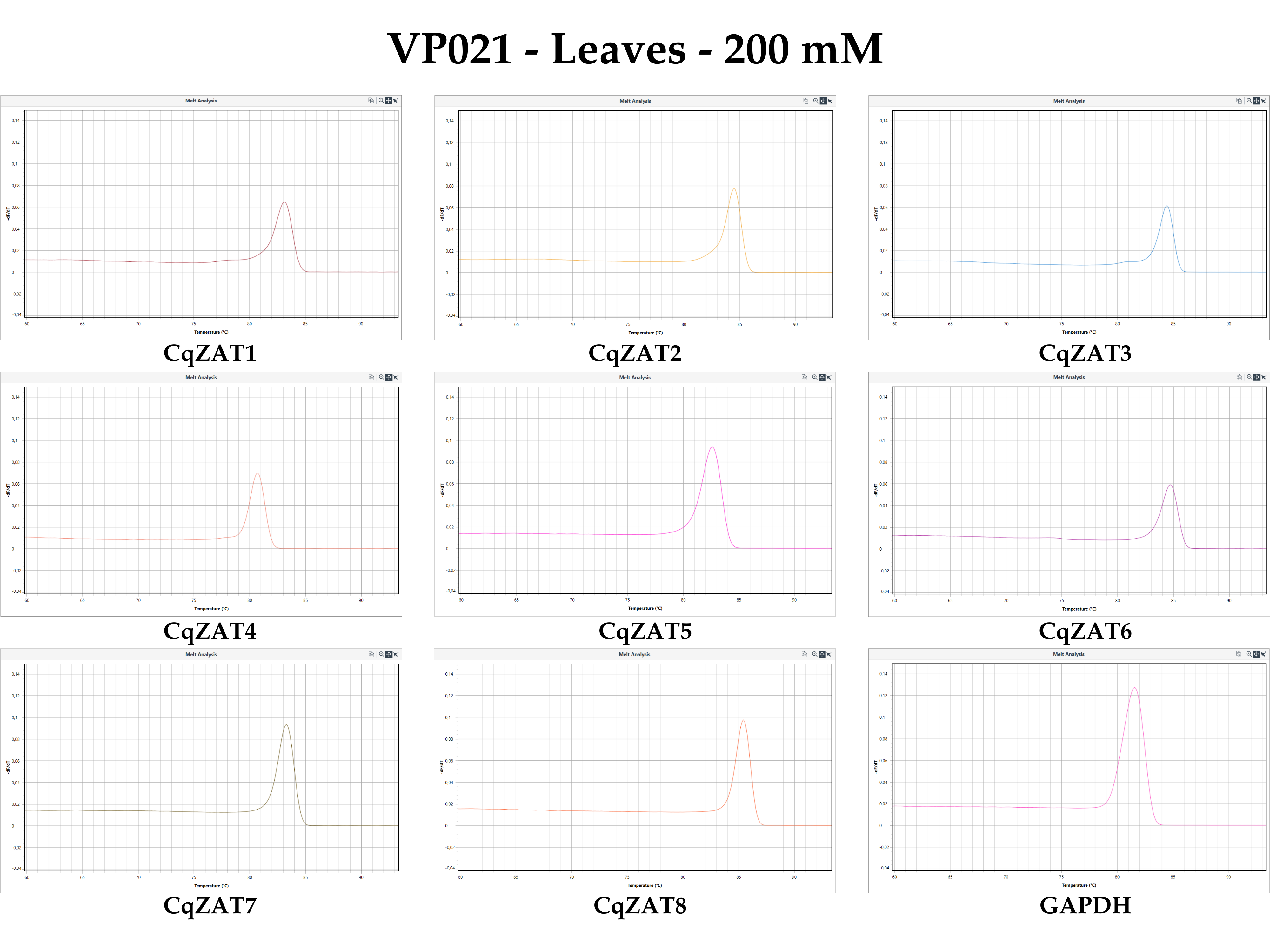

Supplement: Supplementary file 1 [file ijms-26-02570-s001.zip › Figure S10 - VP021 - Leave - 200 mM.png]

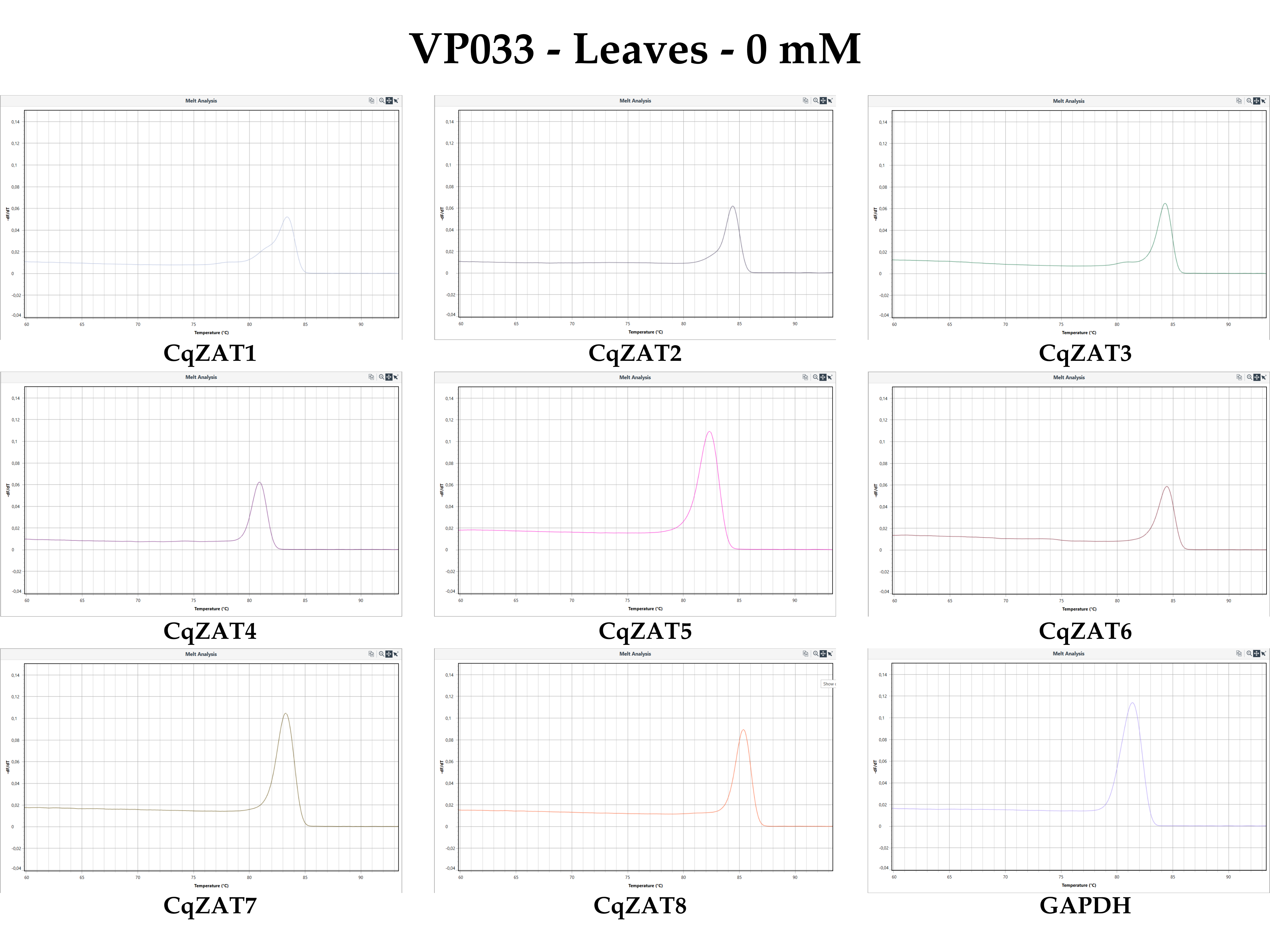

Supplement: Supplementary file 1 [file ijms-26-02570-s001.zip › Figure S11 - VP033 - Leaves - 0 mM.png]

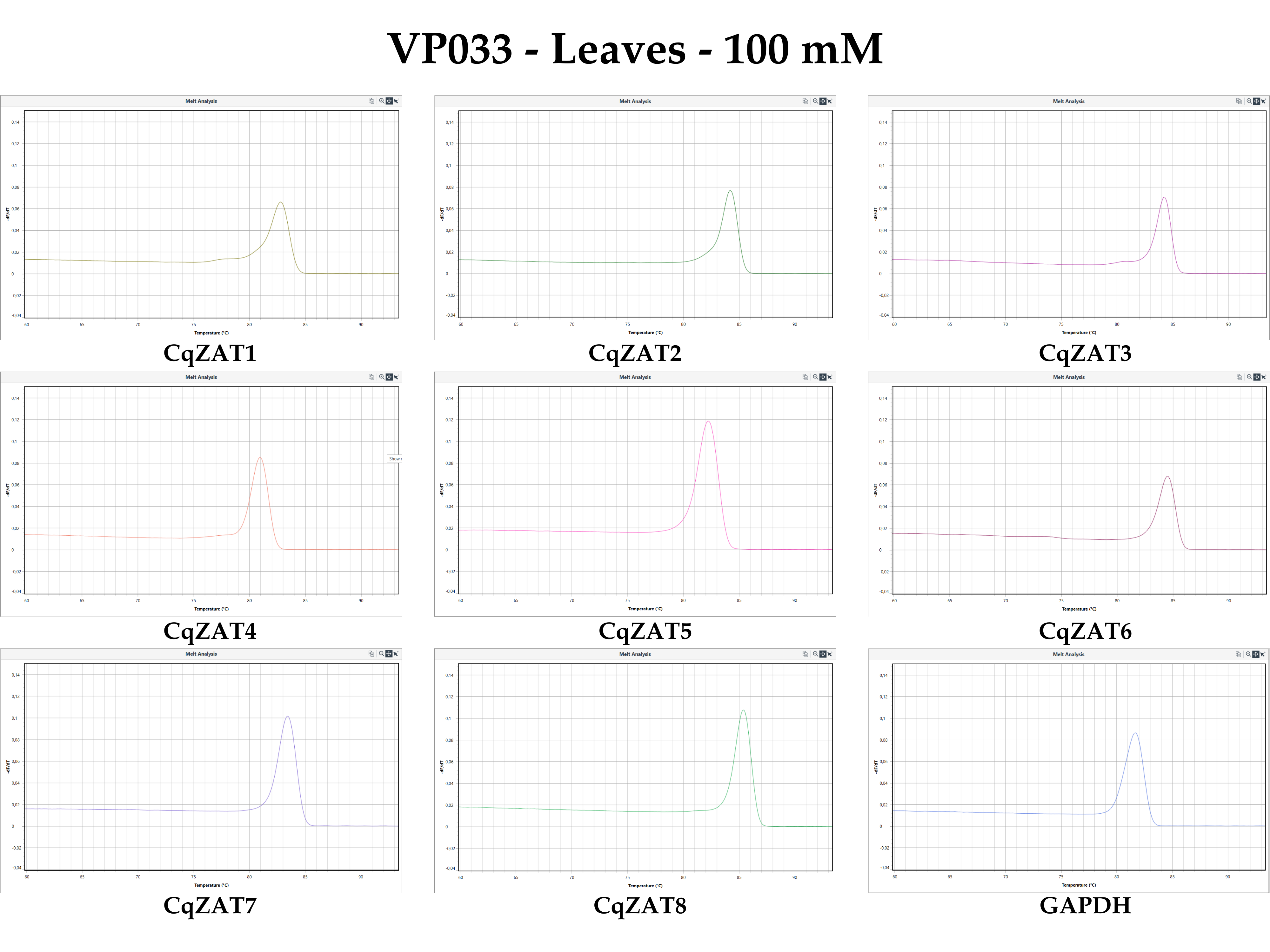

Supplement: Supplementary file 1 [file ijms-26-02570-s001.zip › Figure S12 - VP033 - Leaves - 100 mM.png]

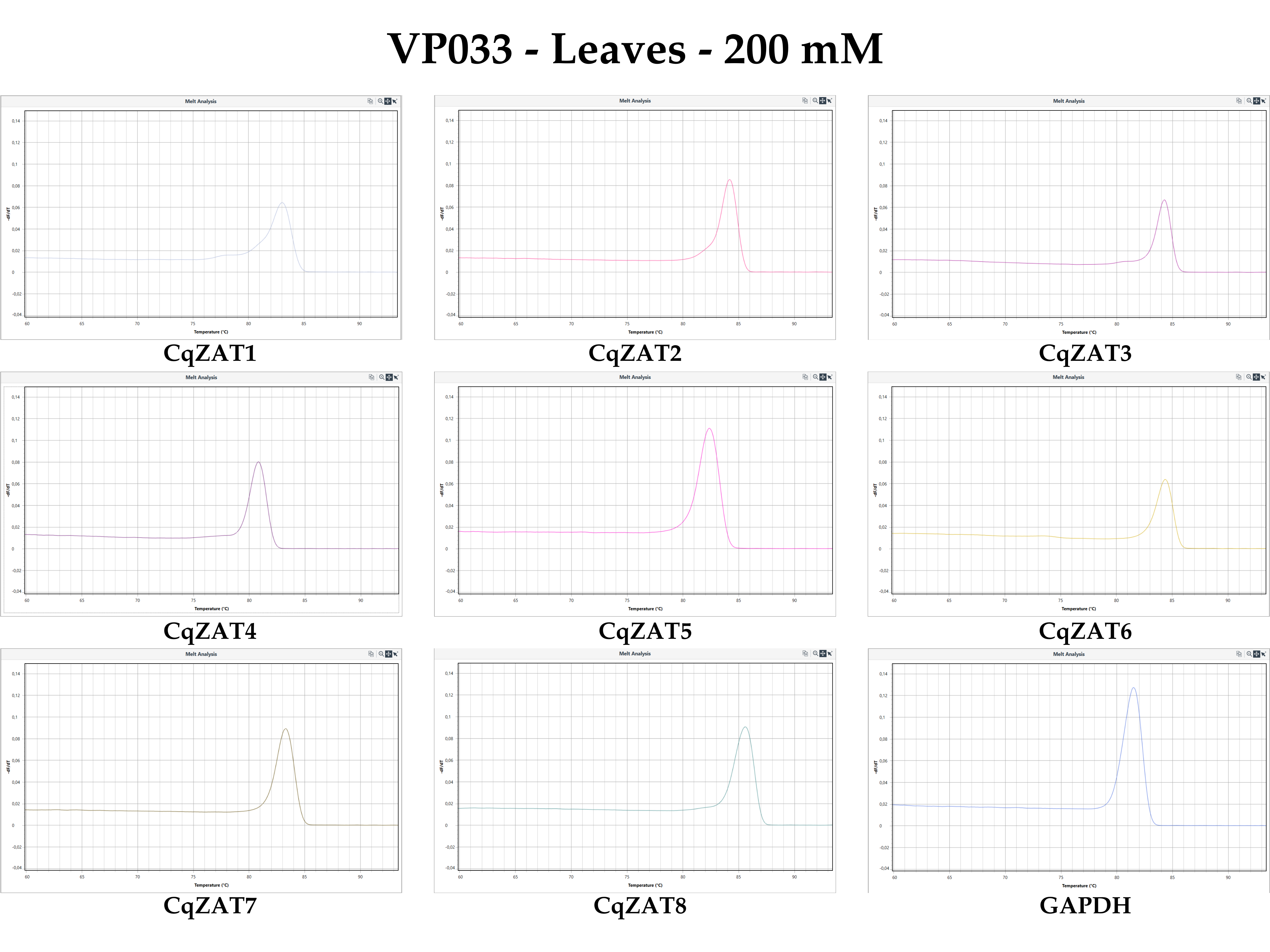

Supplement: Supplementary file 1 [file ijms-26-02570-s001.zip › Figure S13 - VP033 - Leaves - 200 mM.png]

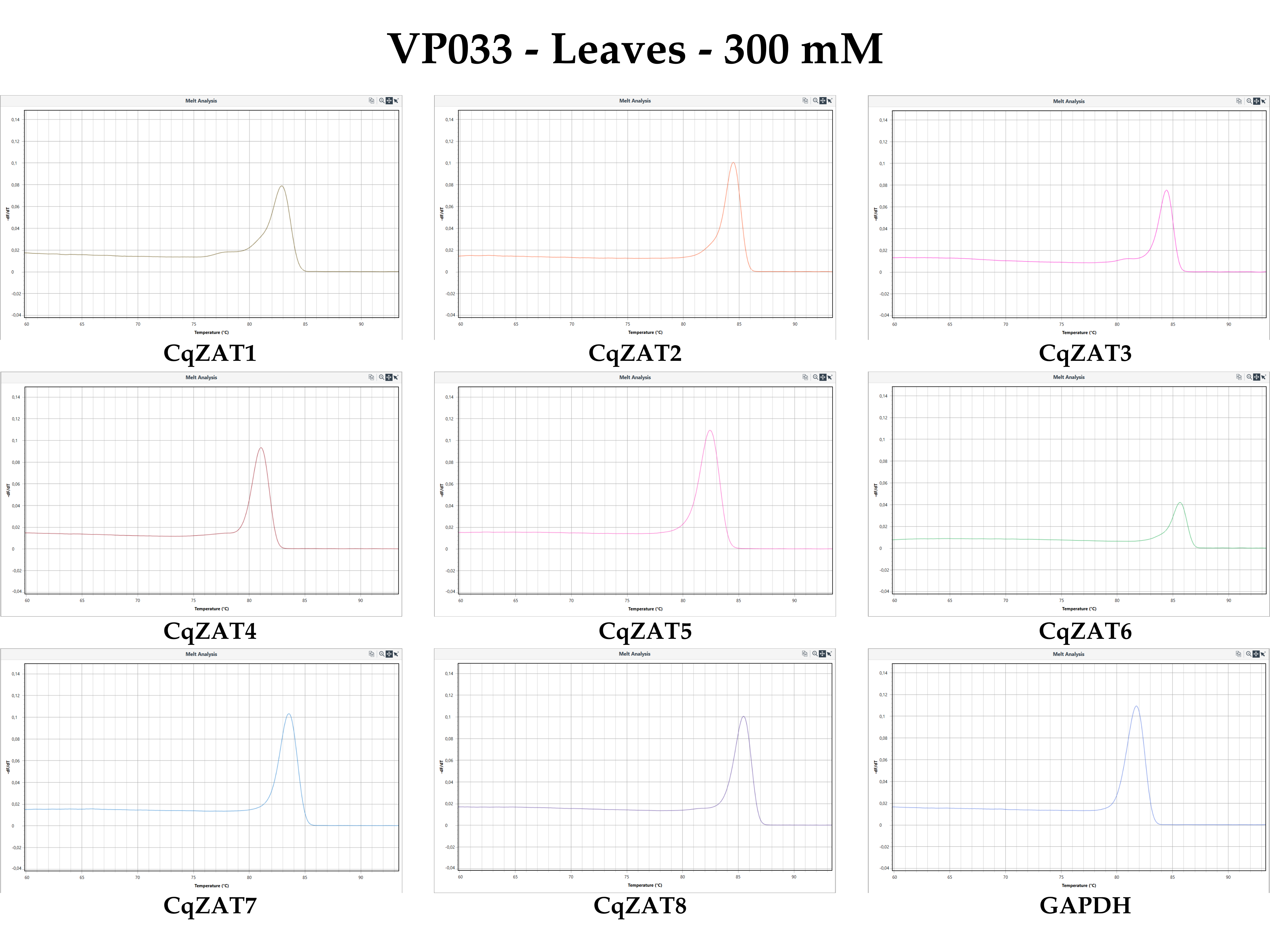

Supplement: Supplementary file 1 [file ijms-26-02570-s001.zip › Figure S14 - VP033 - Leaves - 300 mM.png]

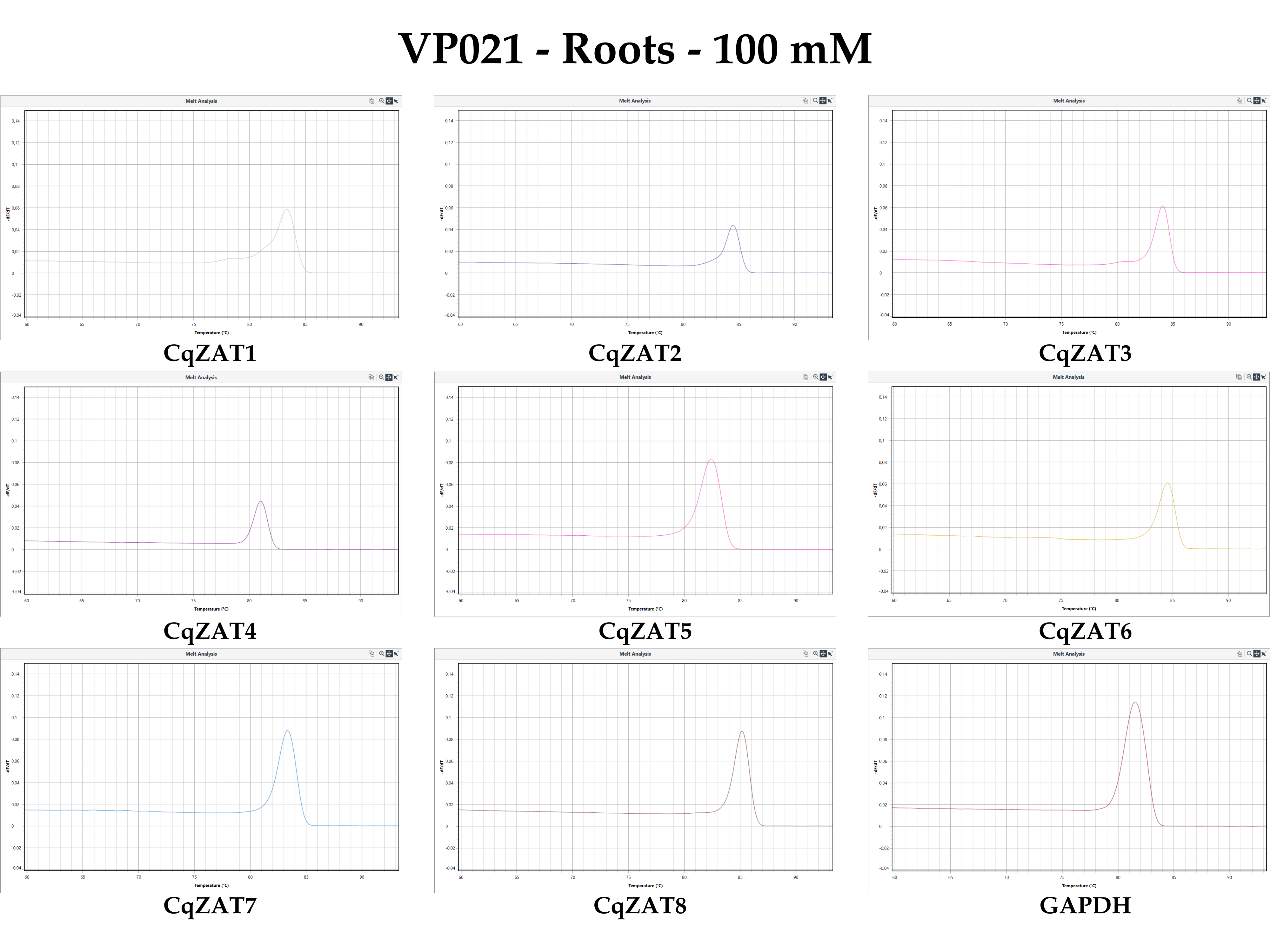

Supplement: Supplementary file 1 [file ijms-26-02570-s001.zip › Figure S2 -VP021 - Root - 100 mM.png]

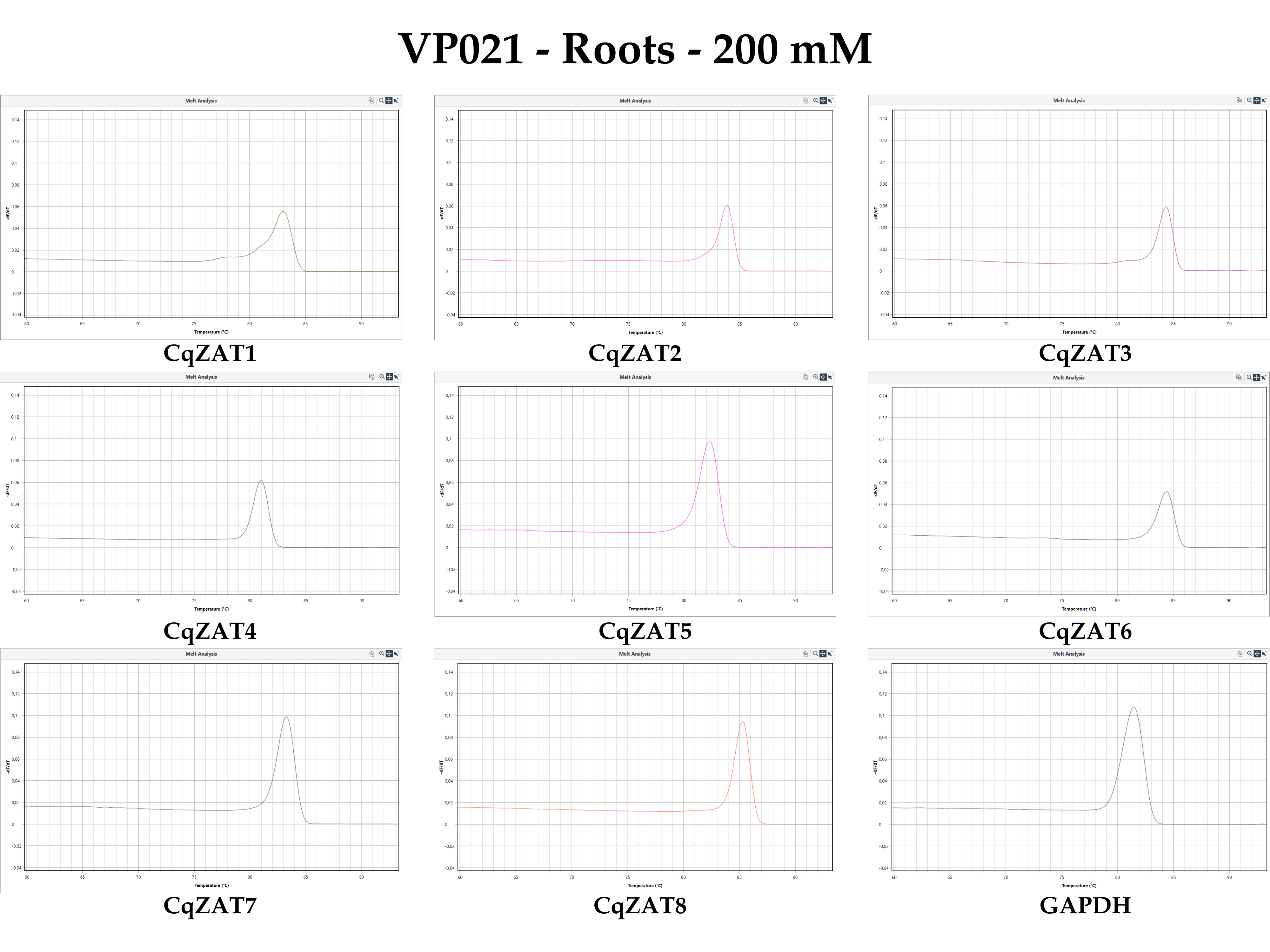

Supplement: Supplementary file 1 [file ijms-26-02570-s001.zip › Figure S3 -VP021 - Root - 200 mM.png]

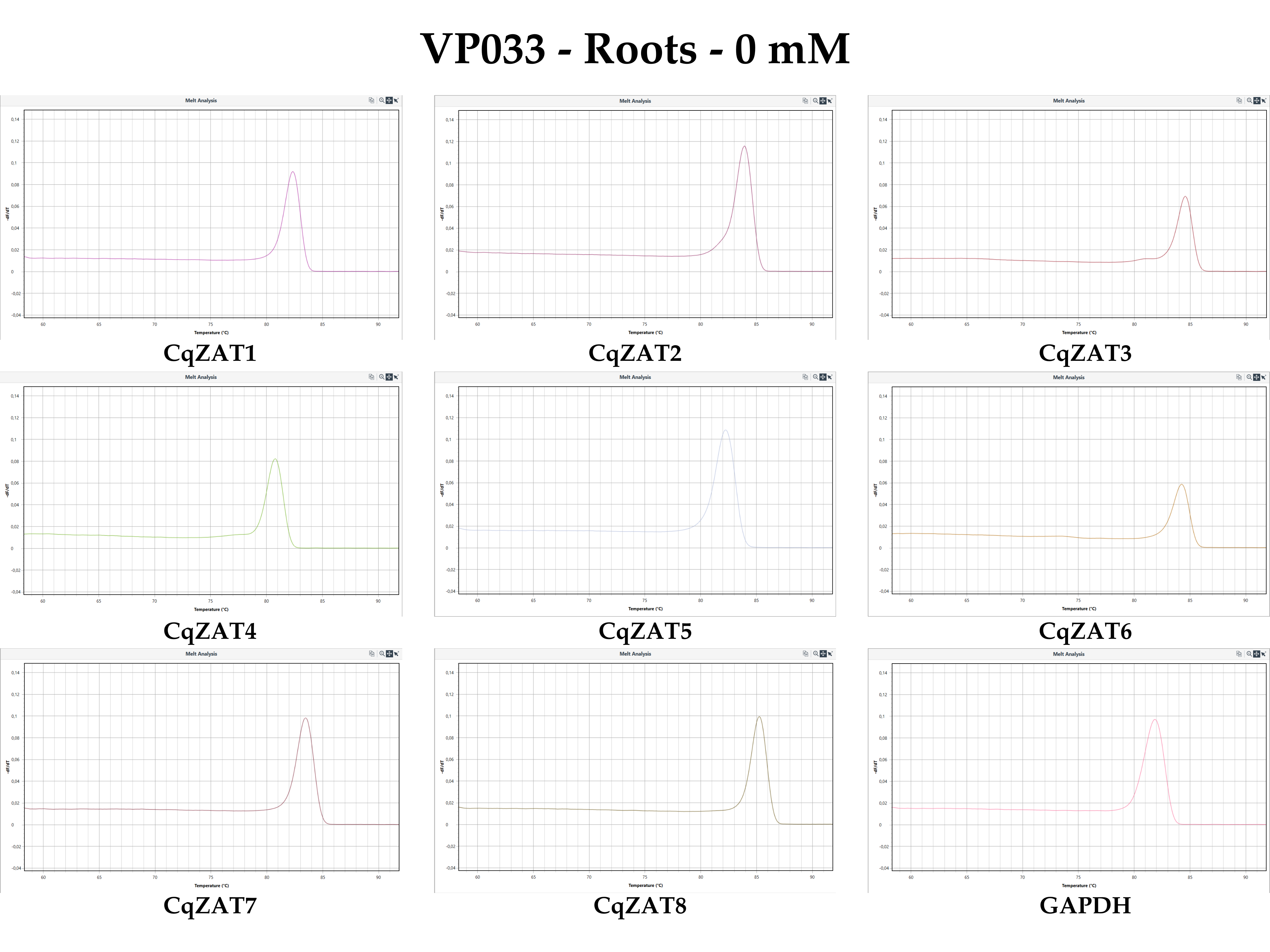

Supplement: Supplementary file 1 [file ijms-26-02570-s001.zip › Figure S4 - VP033 - Root - 0 mM.png]

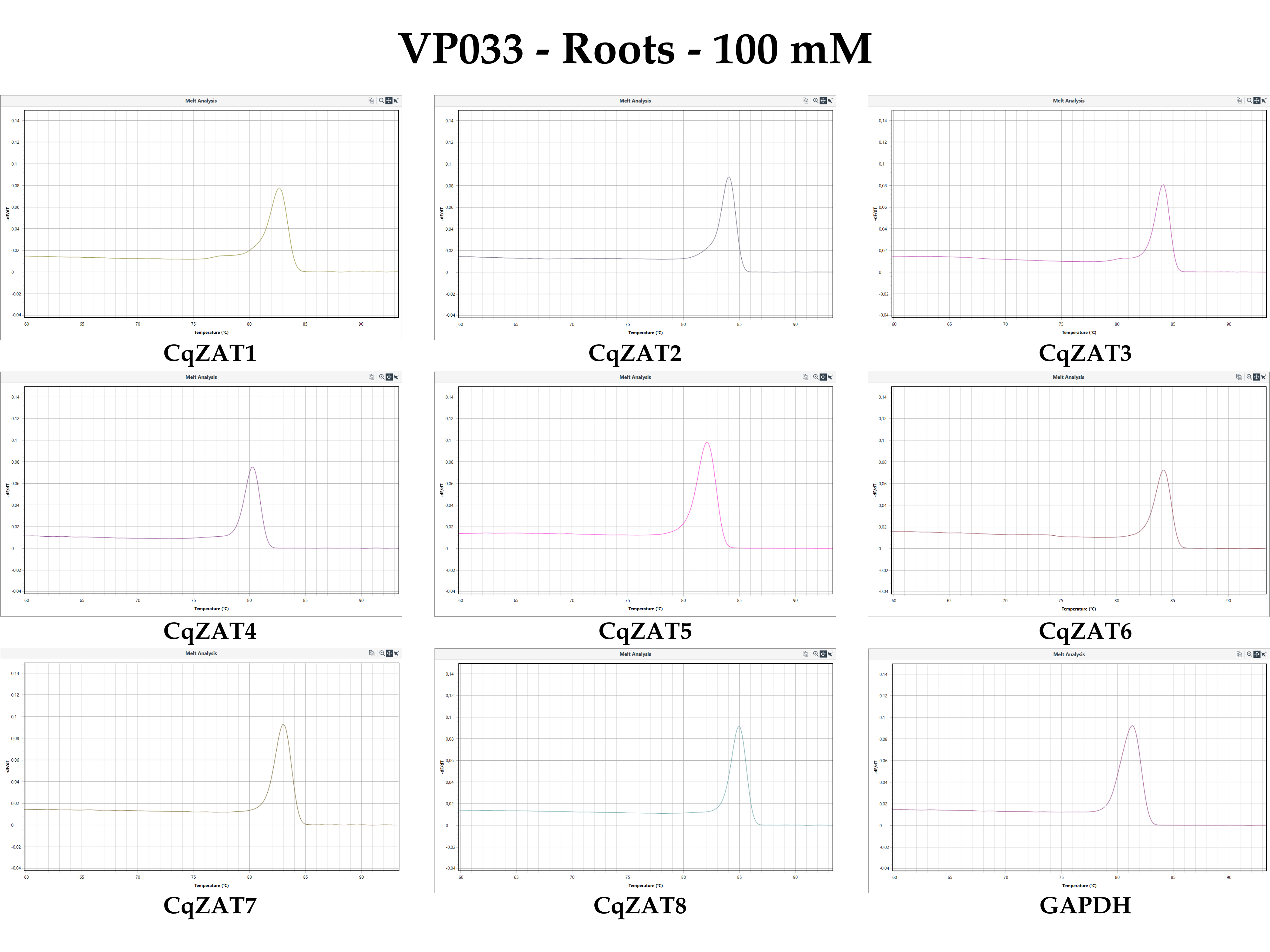

Supplement: Supplementary file 1 [file ijms-26-02570-s001.zip › Figure S5 - VP033 - Root - 100 mM.png]

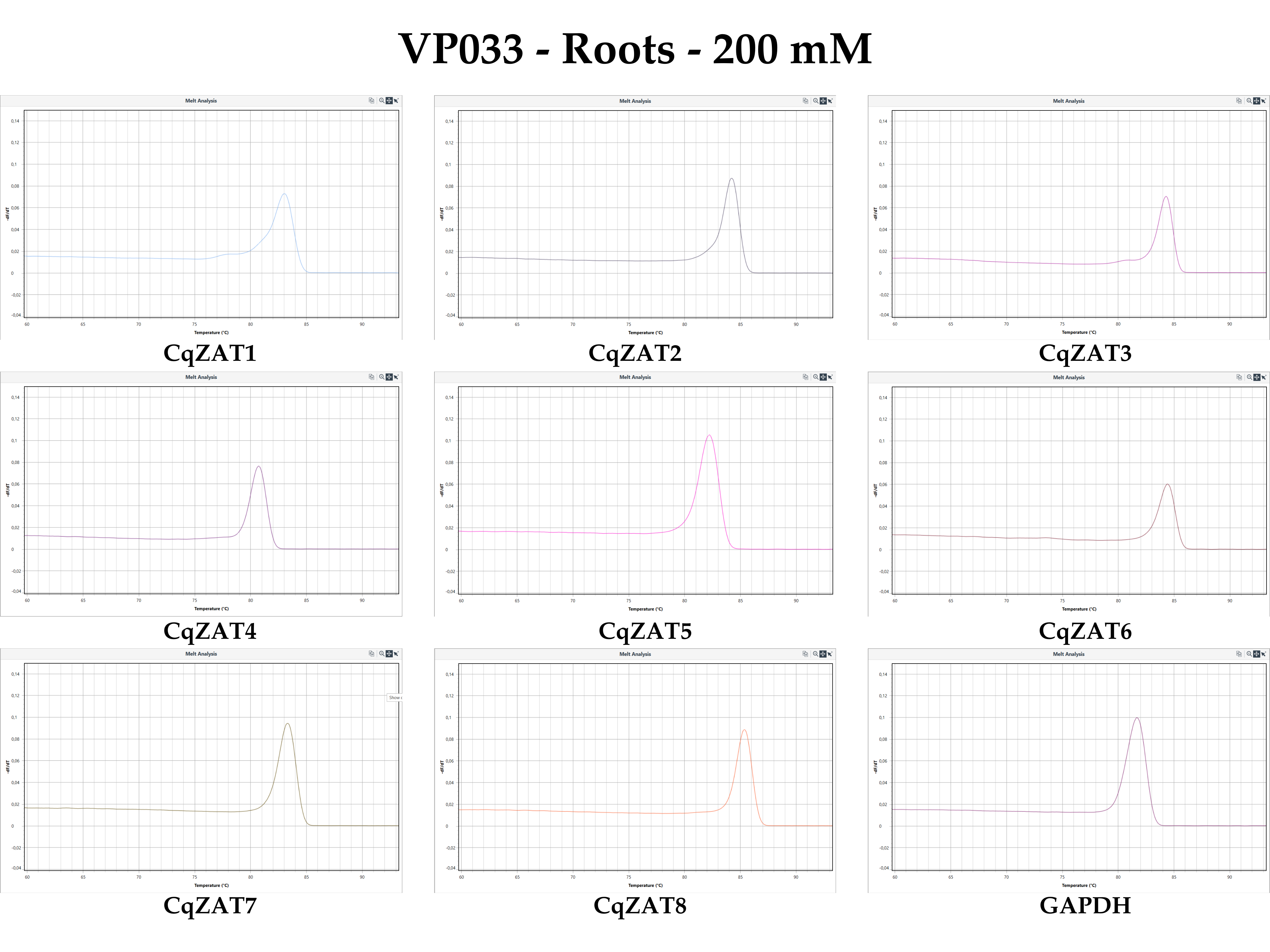

Supplement: Supplementary file 1 [file ijms-26-02570-s001.zip › Figure S6 - VP033 - Root - 200 mM.png]

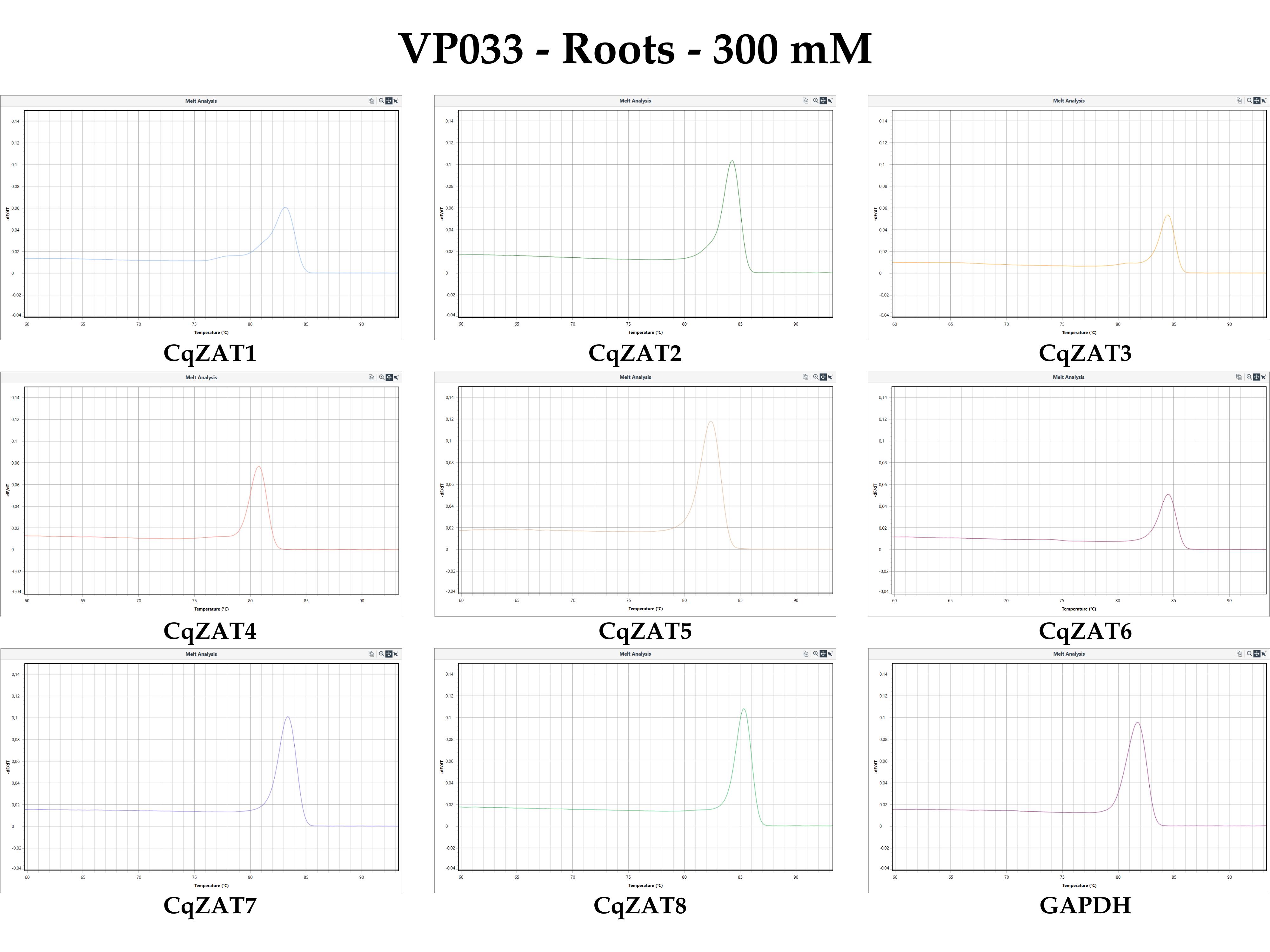

Supplement: Supplementary file 1 [file ijms-26-02570-s001.zip › Figure S7 - VP033 - Root - 300 mM.png]

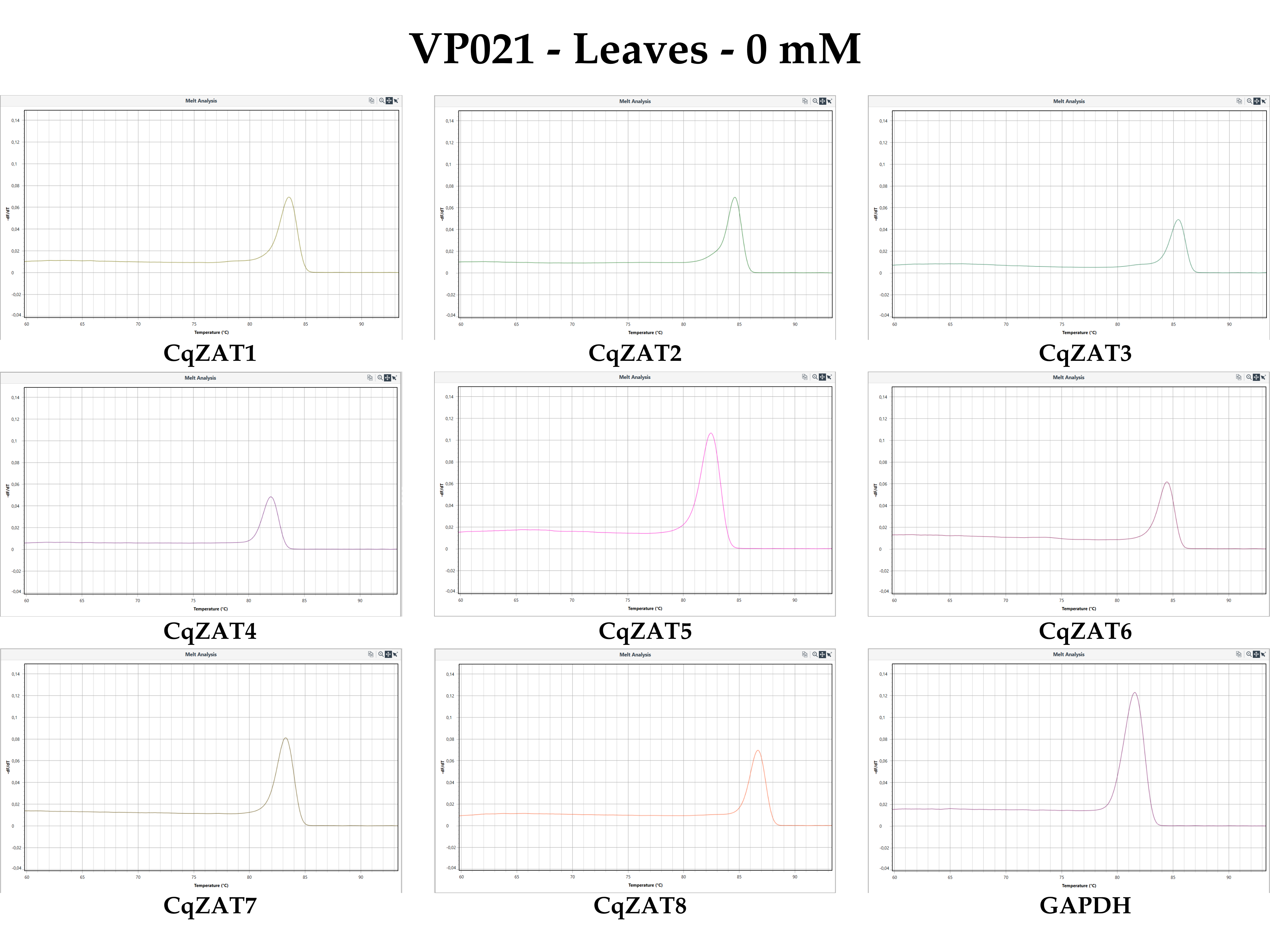

Supplement: Supplementary file 1 [file ijms-26-02570-s001.zip › Figure S8 - VP021 - Leave - 0 mM.png]

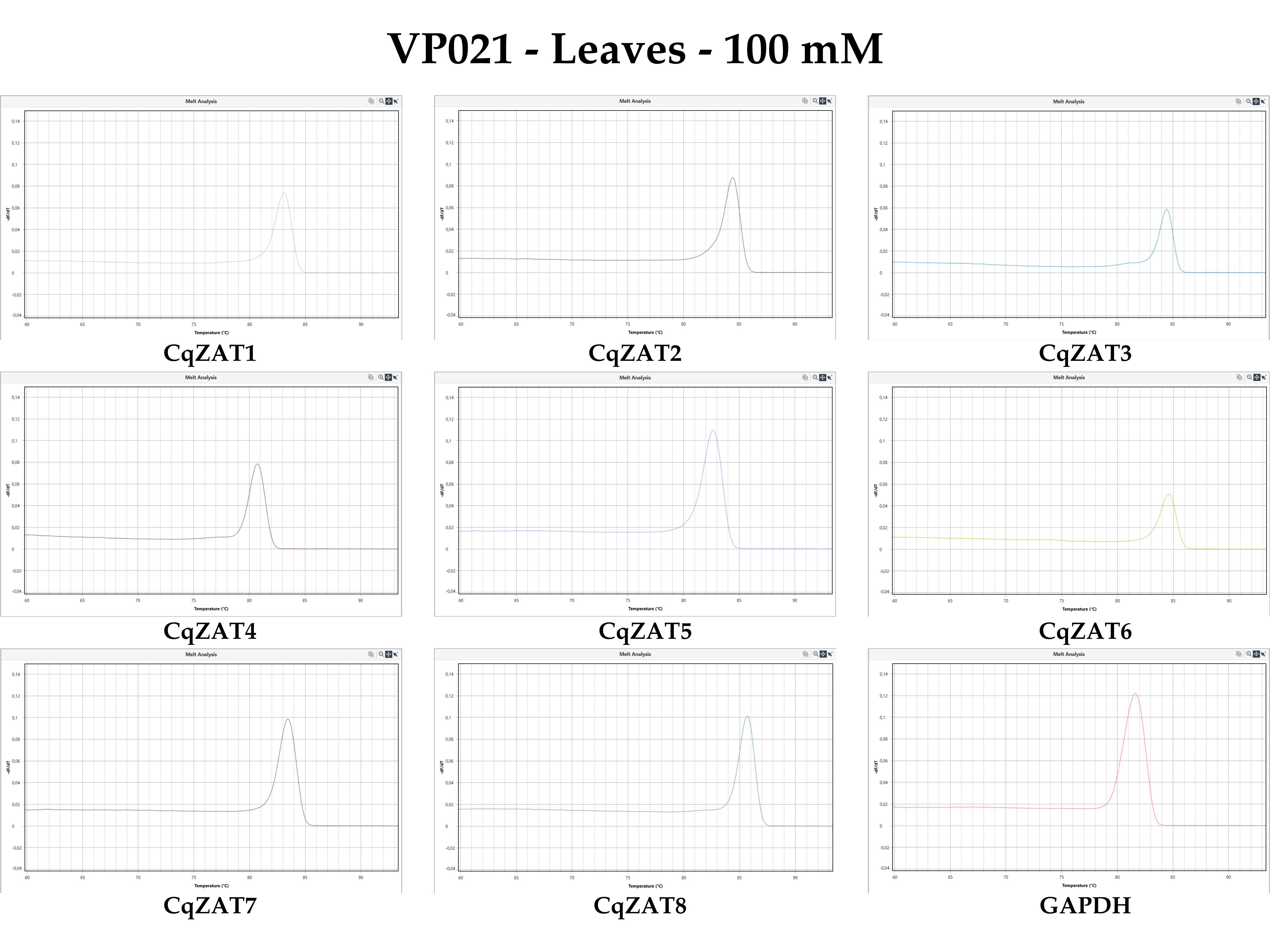

Supplement: Supplementary file 1 [file ijms-26-02570-s001.zip › Figure S9 - VP021 - Leave - 100 mM.png]
